# Peptidylarginine deiminase 2 has potential as both a biomarker and therapeutic target of sepsis

**DOI:** 10.1172/jci.insight.138873

**Published:** 2020-10-15

**Authors:** Yuzi Tian, Shibin Qu, Hasan B. Alam, Aaron M. Williams, Zhenyu Wu, Qiufang Deng, Baihong Pan, Jing Zhou, Baoling Liu, Xiuzhen Duan, Jianjie Ma, Santanu Mondal, Paul R. Thompson, Kathleen A. Stringer, Theodore J. Standiford, Yongqing Li

**Affiliations:** 1 Department of Rheumatology and Immunology, Xiangya Hospital, Central South University, Changsha, Hunan, China.; 2 Department of Surgery, University of Michigan Health System, Ann Arbor, Michigan, USA.; 3 Department of Hepatobiliary Surgery, Xijing Hospital, Xian, Shanxi, China.; 4 Department of Infectious Disease, Second Xiangya Hospital, Central South University, Changsha, Hunan, China.; 5 Trauma Center, Department of Orthopedic and Traumatology, Peking University People’s Hospital, Beijing, China.; 6 Department of Pathology, Loyola University Medical Center, Maywood, Illinois, USA.; 7 Department of Surgery, Davis Heart and Lung Research Institute, The Ohio State University, Columbus, Ohio, USA.; 8 Department of Biochemistry and Molecular Pharmacology, University of Massachusetts Medical School, Worcester, Massachusetts, USA.; 9 Department of Clinical Pharmacy, College of Pharmacy, University of Michigan, Ann Arbor, Michigan, USA.; 10 Division of Pulmonary and Critical Care Medicine, Department of Internal Medicine, University of Michigan Medical Center, Ann Arbor, Michigan, USA.

**Keywords:** Infectious disease, Inflammation, Molecular biology, Molecular pathology

## Abstract

Peptidylarginine deiminases (PADs) are a family of calcium-dependent enzymes that are involved in a variety of human disorders, including cancer and autoimmune diseases. Although targeting PAD4 has shown no benefit in sepsis, the role of PAD2 remains unknown. Here, we report that PAD2 is engaged in sepsis and sepsis-induced acute lung injury in both human patients and mice. *Pad2^–/–^* or selective inhibition of PAD2 by a small molecule inhibitor increased survival and improved overall outcomes in mouse models of sepsis. *Pad2* deficiency decreased neutrophil extracellular trap (NET) formation. Importantly, *Pad2* deficiency inhibited Caspase-11–dependent pyroptosis in vivo and in vitro. Suppression of PAD2 expression reduced inflammation and increased macrophage bactericidal activity. In contrast to *Pad2^–/–^*, *Pad4* deficiency enhanced activation of Caspase-11–dependent pyroptosis in BM-derived macrophages and displayed no survival improvement in a mouse sepsis model. Collectively, our findings highlight the potential of PAD2 as an indicative marker and therapeutic target for sepsis.

## Abstract 2

Peptidylarginine deiminases 2 (PAD2) regulates neutrophil extracellular trap (NET) formation in sepsis and sepsis-induced acute lung injury.

## Introduction

Sepsis is a life-threatening organ dysfunction caused by a dysregulated host response to infection (1). As of 2017, sepsis accounted for 11 million deaths worldwide (2). Many efforts have focused on developing therapeutic targets, but relatively effective treatments for sepsis are lacking.

In defense against microbial infection, innate immune cells are activated, causing neutrophil extracellular trap (NET) formation (NETosis, a programmed neutrophil death) and macrophage pyroptosis (an inflammatory cell death). NETosis sequesters bacteria for pathogen inactivation (3), and pyroptosis is an antimicrobial response that mainly takes place in macrophages. Although uncontrolled immune cell death has recently been considered as a significant contributing factor to sepsis pathogenesis (4–6), it is presently unknown whether manipulation of the regulation of NETosis and/or pyroptosis influences sepsis progression.

Peptidylarginine deiminases (PADs) are a family of calcium-dependent enzymes that promote the conversion of the basic amino acid residue arginine to a neutral residue citrulline. The 5 PAD isozymes (PAD1–PAD4 and PAD6) share overlapping substrates. Due to their function in citrullinated proteins, PADs are involved in a variety of physiological processes, including gene expression, immune response, and cell signaling (7). Among the PADs, PAD2 and PAD4 are highly expressed in neutrophils (8) and macrophages (9), and they are widely involved in immune-related activity. PAD4 is involved in the signaling pathway of NETosis (10), but *Pad4*-deficient mice are not protected in models of sepsis (11). Interestingly, pan-PAD inhibitors have been shown to improve survival in a mouse model of sepsis (12), suggesting that PADs other than PAD4 may play a key role in pathogenesis of sepsis. Furthermore, recent studies have shown that PAD2 inhibition is beneficial in several animal models of diseases, including lupus, colitis, cancer, and hemorrhagic shock (13, 14). However, the relationship between PAD2 and sepsis is still elusive.

In this study, we assessed the expression of PAD2 in human septic patients. Using *Pad2^–/–^* mice or mice treated with a selective PAD2 inhibitor, AFM32a, we evaluated the impact of PAD2 on survival and outcomes in animal models of sepsis. We also determined the effects of PAD2 on neutrophil NET formation and macrophage Caspase-11–dependent pyroptosis (noncanonical pyroptosis).

## Results

### PAD2 protein is increased in patients with sepsis and in a mouse model of sepsis.

**Figure 1:**
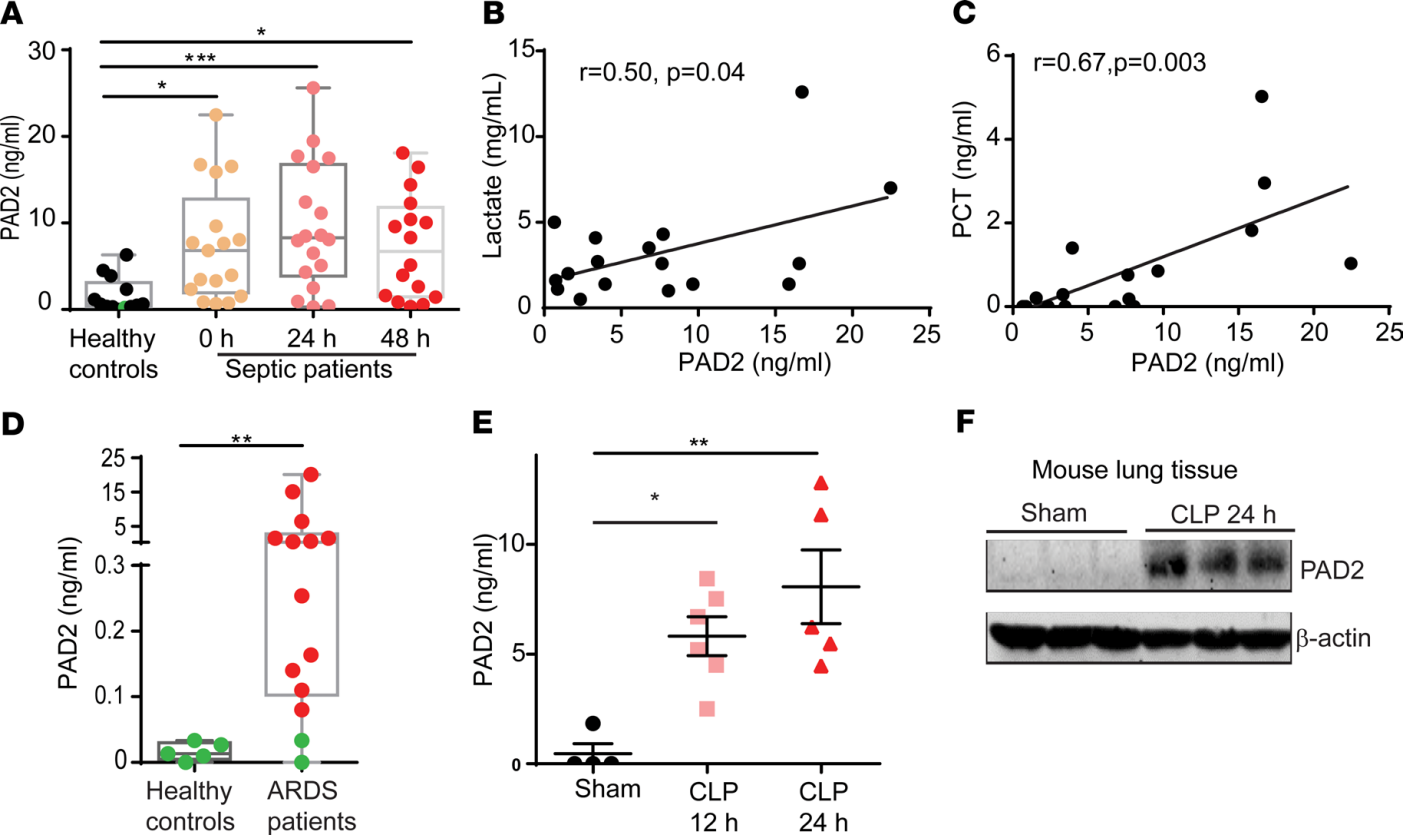
PAD2 protein is increased in patients with sepsis and a murine model of sepsis. (**A**) Levels of serum PAD2 protein in healthy controls and patients with sepsis. Serum samples from patients with sepsis collected at enrollment, 24 hours, and 48 hours (*n* = 13–18/group) were assayed. (**B**) Correlation of serum concentrations of PAD2 protein and lactate in patients with sepsis at enrollment (*n* = 17). (**C**) Correlation of serum concentrations of PAD2 protein and procalcitonin (PCT) in patients with sepsis at enrollment (*n* = 17). The line in the graphs represents the linear fit. (**D**) Levels of PAD2 protein in bronchoalveolar lavage fluid from healthy controls and patients with ARDS (*n* = 5 for healthy controls and *n* = 14 for patients with sepsis-induced ARDS). (**E**) Levels of serum PAD2 protein in sham and CLP mice at 12 hours and 24 hours (*n* = 4–6/group). (**F**) Western blot results show the expression of PAD2 protein in mouse lung tissue with or without CLP (*n* = 3). Nonnormality data are expressed as minimal to maximal value with quantile range (**A** and **D**). Data in **E** are expressed as mean ± SEM. Data with 3 or more groups were analyzed by Kruskal-Wallis test with Dunnett’s multiple comparisons test (**A**) or 1-way ANOVA with Bonferroni’s multiple comparisons test (**E**). Two groups were analyzed using Mann-Whitney *U* test (**D**). Correlations between concentrations of PAD2 and lactate or PCT were analyzed using Pearson correlation model. *P < 0.05, **P < 0.01, ***P < 0.001. The green dots in **A** and **D** were values below the lower limit of quantification of the ELISA kit but based on standard curve back calculation.

To investigate whether PAD2 protein is upregulated in sepsis, we measured PAD2 protein in serum samples collected from patients with sepsis at study enrollment and at 24 and 48 hours after enrollment. The serum of healthy volunteers served as a negative control. Circulating PAD2 was increased in patients with sepsis compared with healthy controls. This increase was significant and persisted for about 48 hours (Figure 1A).

Further analysis showed that the serum concentration of PAD2 in patients was positively correlated to lactate (*r* = 0.5, *P* = 0.04) (Figure 1B) and procalcitonin (PCT) levels (*r* = 0.67, *P* = 0.003) (Figure 1C). Since acute respiratory distress syndrome (ARDS) is a common complication in sepsis, we measured levels of PAD2 in bronchoalveolar lavage fluid (BALF) that was collected from patients within 7 days of the diagnosis of sepsis-induced ARDS. Consistent with serum samples, PAD2 protein in BALF from the ARDS patients was also significantly increased compared with the healthy control group (Figure 1D).

Next, we determined whether PAD2 protein was elevated in a murine model of cecal ligation and puncture–induced (CLP-induced) lethal sepsis. Consistent with our findings from patients with sepsis, PAD2 was also upregulated in serum and lung tissue after CLP (Figure 1, E and F). Our results suggest that PAD2 is involved in the pathogenesis of human sepsis and may participate in the underlying mechanisms that lead to sepsis-induced end organ damage.

### Pad2 deficiency improves survival in the mouse model of CLP-induced sepsis.

**Figure 2:**
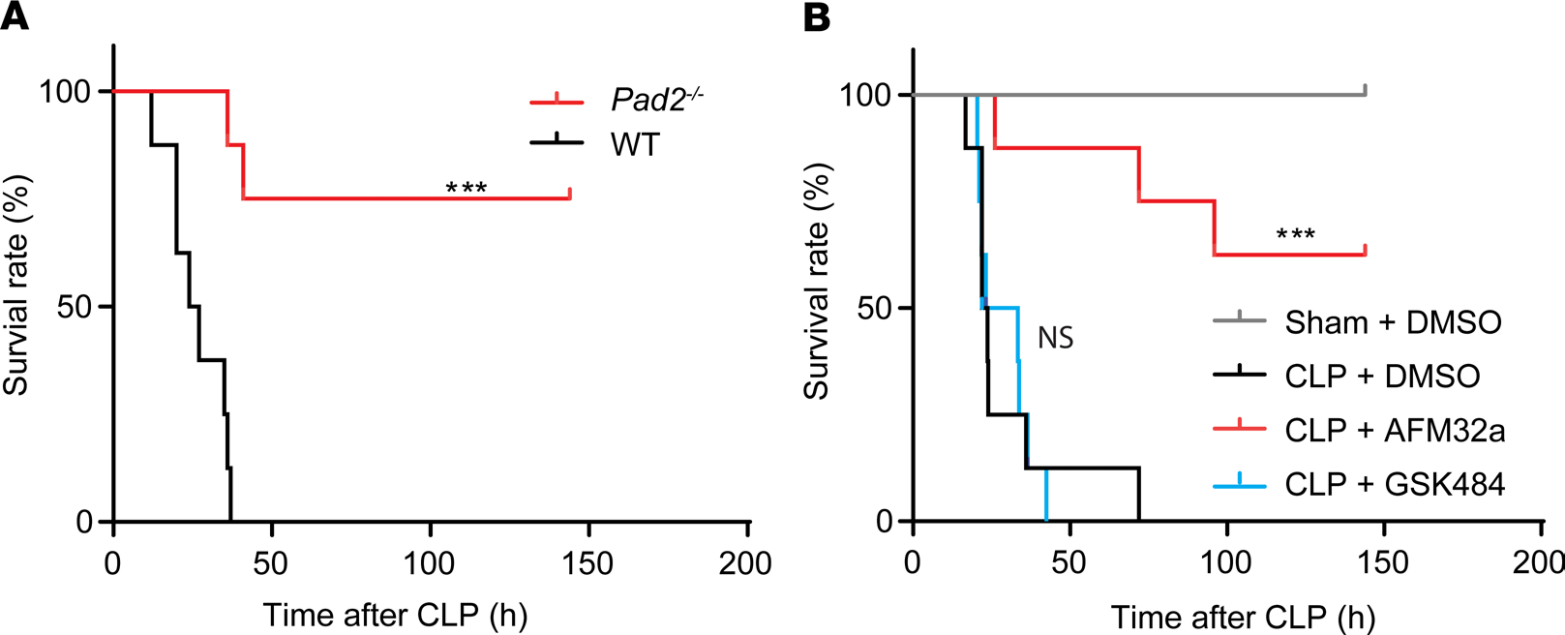
Absence of the *Pad2* gene or inhibition of PAD2 improves survival in a CLP murine model of sepsis. (**A**) Kaplan-Meier survival curves of WT and *Pad2^–/–^* mice subjected to CLP (*n* = 8/group). (**B**) Kaplan-Meier survival curves of WT mice subjected to CLP that were pretreated with the PAD2 selective inhibitor AFM32a, PAD4 selective inhibitor GSK484, or DMSO vehicle (*n* = 8/group). Values are expressed as survival percentage. ***P < 0.001 by log-rank test.

To understand the role of PAD2 in sepsis, we first assessed whether *Pad2* gene KO would impact mortality in the murine CLP model. Compared with WT mice, the survival rate of *Pad2^–/–^* mice was significantly improved for both sexes (Figure 2A and Supplemental Figure 1; supplemental material available online with this article; https://doi.org/10.1172/jci.insight.138873DS1). In addition, WT mice treated with the PAD2 selective inhibitor AFM32a had a significant increase in survival compared with vehicle controls (Figure 2B). Our results indicate that inhibition of PAD2 expression can significantly improve survival in the murine model of CLP-induced sepsis.

### Pad2^–/–^ enhances bacterial clearance and improves organ functions in the murine CLP-induced sepsis model.

**Figure 3:**
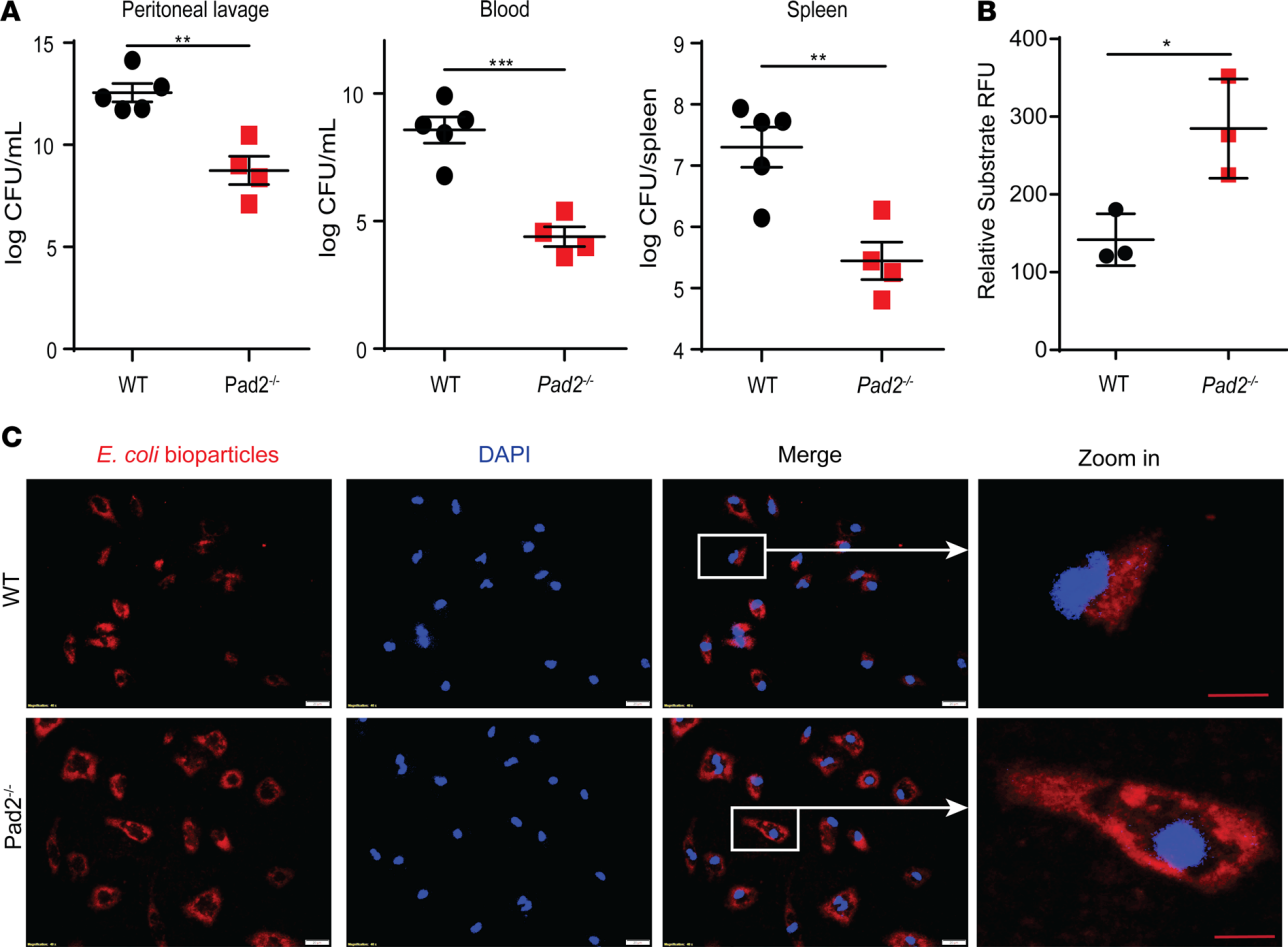
Absence of the *Pad2* gene improves bacterial clearance in a CLP murine model of sepsis and enhances phagocytosis in BM-derived macrophages (BMDMs). WT and *Pad2^–/–^* mice were subjected to CLP. (**A**) Bacterial loads in peritoneal lavage, blood, and spleen (*n* = 4–5/group) after 24 hours. BMDMs were isolated from WT and *Pad2^–/–^* mice and incubated with 1 mg/mL pHrodo Red *E*. *coli* BioParticles. (**B** and **C**) Fluorescence signal of engulfed *E*. *coli* bioparticles in peritoneal macrophage (**B**) and its representative images (**C**). Values are relative fluorescence units (RFU), calculated from 3 wells per group. The results in **B** and **C** are representative of 3 independent experiments. Data are expressed as mean ± SEM. Data with 2 groups were analyzed using an unpaired 2-tailed Student’s *t* test. *P < 0.05, **P < 0.01, ***P < 0.001. White scale bar: 20 μm. Red scale bar: 10 μm.

To further evaluate outcomes in the murine CLP model, several pathological parameters were assessed. Compared with WT mice, the bacterial CFU in the abdominal cavity of *Pad2^–/–^* mice were significantly decreased by more than 1000-fold at 24 hours following CLP (Figure 3A). Decreased bacterial dissemination in *Pad2^–/–^* mice was also evident in the blood and spleen with a nearly 10,000- and 100-fold decline in CFU compared with WT control animals, respectively. To determine whether the reduced bacterial load may partly result from the altered activity of phagocytes (macrophages and neutrophils), we assessed phagocytic function of *Pad2-*deficient BM-derived macrophage (BMDMs), peritoneal macrophages, and neutrophils. Compared with WT macrophages, *Pad2-*deficient BMDMs and peritoneal macrophages displayed enhanced phagocytosis (Figure 3, B and C; and Supplemental Figure 2, A and B), as shown by the significantly increased uptake of *E*. *coli* bioparticles. Neutrophils, however, showed similar phagocytosis capacity in WT and *Pad2^–/–^* mice (Supplemental Figure 2, C and D).

**Figure 4:**
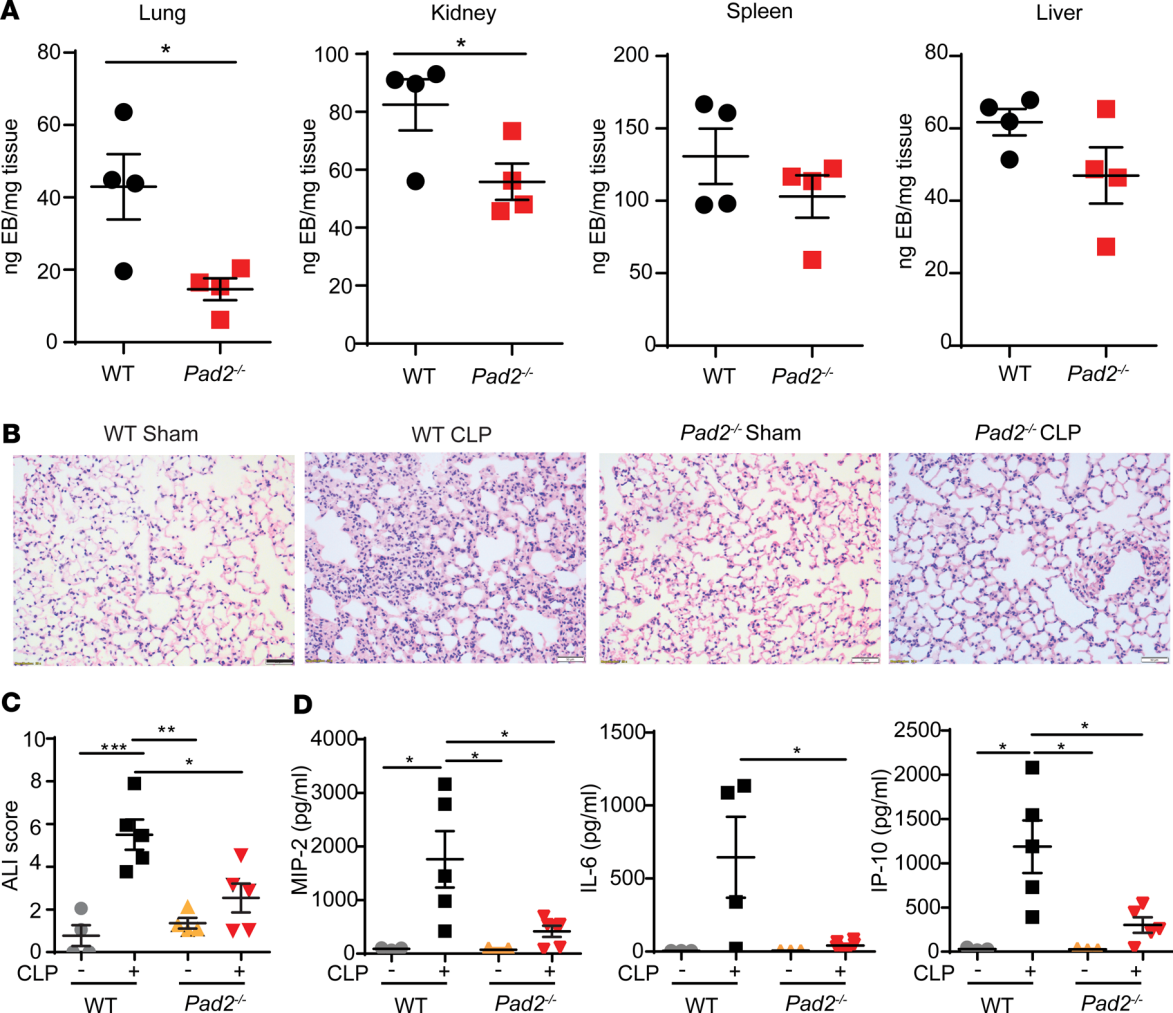
Absence of the *Pad2* gene attenuates vascular permeability and ALI in a CLP murine model of sepsis. WT and *Pad2^–/–^* mice were subjected to CLP. (**A**) Vascular permeability in lung, kidney, spleen, and liver after 24 hours (*n* = 4/group) as measured by the weight of EB dye in tissue. (**B**) Representative histology images of murine lungs following H&E staining and (*n* = 4–5/group). (**C** and **D**) ALI score (**C**) and concentrations of MIP-2, IL-6, and IP-10 (**D**) in the bronchoalveolar lavage fluid (*n* = 3-6/group). Data are expressed as mean ± SEM. Data with 2 groups were analyzed using an unpaired 2-tailed Student’s *t* test (**A**), and 3 or more groups were analyzed by 2-way ANOVA with Bonferroni’s multiple comparisons test (**C** and **D**). *P < 0.05, **P < 0.01, ***P < 0.001. Scale bar: 50 μm.

It is known that enhanced permeability of the endothelial barrier and subsequent microvascular leakage are signs of the advanced stages of sepsis (15). Therefore, we examined the microvascular permeability of different organs. *Pad2^–/–^* significantly reduced CLP-induced permeability in lung and kidney, and it showed a trend toward the decreased permeability in spleen and liver (Figure 4A), suggesting that KO of the *Pad2* gene could ameliorate sepsis-induced organ dysfunction by reducing microvascular permeability.

Given that the main cause of death in sepsis is dysfunction of vital organs — brought about by syndromes such as ARDS (16), a severe form of acute lung injury (ALI) — we performed H&E staining of pulmonary tissues and evaluated the pathological changes of ALI 24 hours after CLP. As shown in Figure 4B, the WT control mice displayed normal histology, while the lungs from WT-CLP group exhibited obvious inflammatory changes (e.g., inflammatory infiltrates, alveolar hemorrhage, pulmonary congestion, edema, and thickening of the alveolar wall). However, the sepsis-induced ALI was significantly attenuated in the *Pad2^–/–^* group, as shown by the significantly reduced ALI score (Figure 4C). IHC experimentation further demonstrated the extensive infiltration of neutrophils and macrophages in lung tissues after CLP. *Pad2* deficiency significantly reduced neutrophil infiltration, but the numbers of macrophages were not changed between the WT and *Pad2^–/–^* groups (Supplemental Figure 3). Moreover, proinflammatory cytokines such as macrophage inflammatory protein 2 (MIP-2), IL-6, and IFN-γ–induced protein 10 (IP-10) were dramatically decreased in BALF of *Pad2^–/–^* mice (Figure 4D). Overall, the results of survival, bacterial loading, vascular permeability, and ALI score strongly indicate that PAD2 is vital for sepsis pathogenesis and that its inhibition could serve as a potential therapeutic target for sepsis.

### Pad2^–/–^ decreases NET formation.

**Figure 5:**
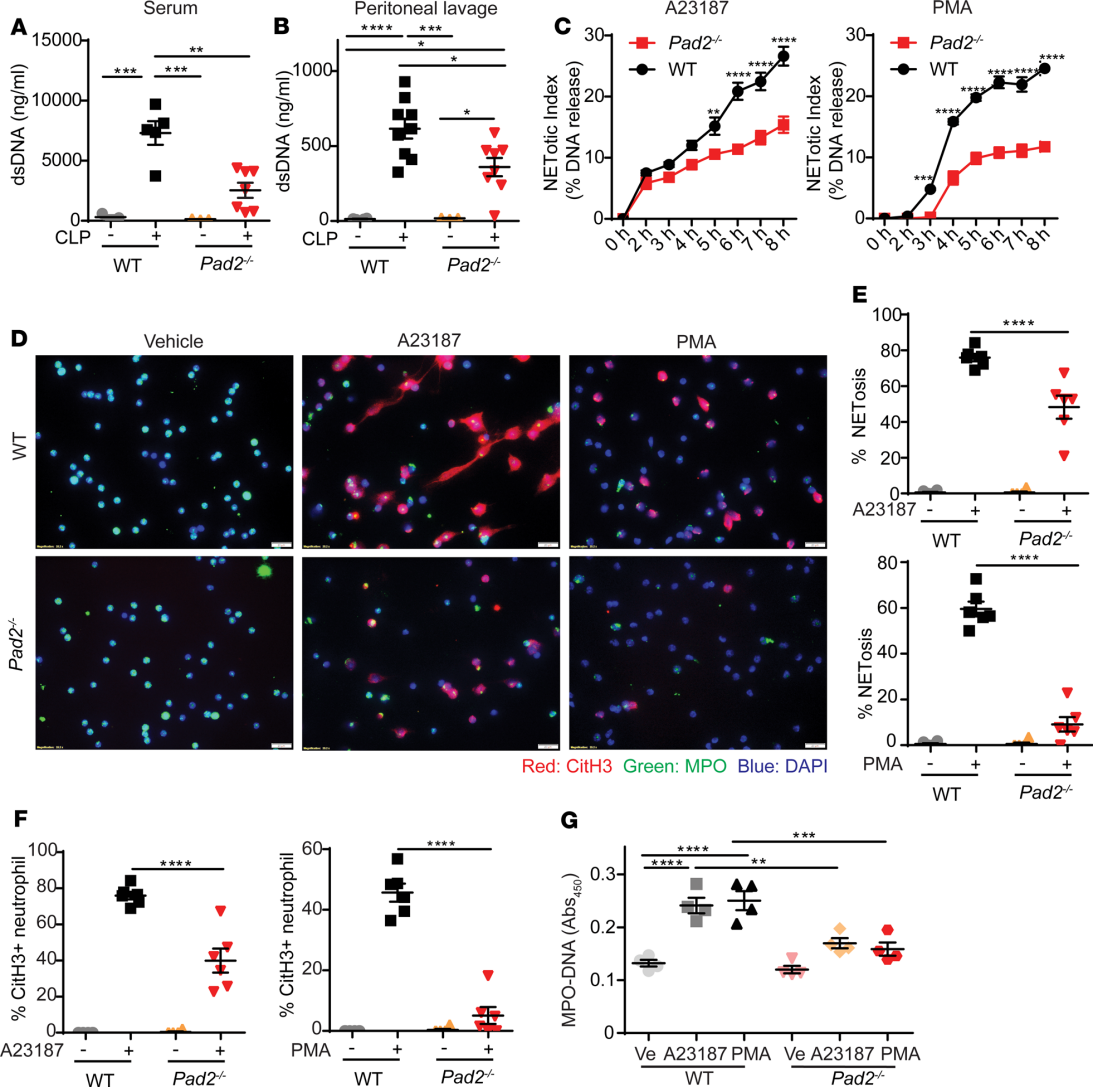
Absence of the *Pad2* gene inhibits NET formation both in vivo and in vitro. WT and *Pad2^–/–^* mice were subjected to CLP. (**A** and **B**) The concentrations of dsDNA in mouse serum (*n* = 3 for WT mice without CLP group, *n* = 5 for WT mice with CLP group, *n* = 3 for *Pad2^–/–^* mice without CLP group, *n* = 7 for *Pad2*^–/–^ mice with CLP group) (**A**) and peritoneal lavage (*n* = 4 for WT mice without CLP group, *n* = 9 for WT mice with CLP group, *n* = 3 for *Pad2^–/–^* mice without CLP group, and *n* = 8 for *Pad2^–/–^* mice with CLP group) (**B**) after 24 hours. BM neutrophils were isolated from WT and *Pad2^–/–^* mice and were treated with A23187 or PMA. (**C**) NET formation in response to calcium ionophore and PMA in WT and *Pad2^–/–^* neutrophils at different time points (*n* = 3–5/group). (**D**) Representative immunofluorescence images of the NET formation in WT and *Pad2^–/–^* neutrophils after vehicle, Ca^2+^ ionophore (A23187), or PMA treatment for 3.5 hours. (**E** and **F**) Quantification results of immunofluorescence images (*n* = 6/group). (**G**) Levels of myeloperoxidase-DNA (MPO-DNA) in cell supernatant of WT and *Pad2^–/–^* neutrophils after vehicle, A23187or PMA treatment for 3.5 hours. Results are representative of 2 independent experiments (**C**–**G**). Data are expressed as mean ± SEM. Data were analyzed by 2-way ANOVA with Bonferroni’s multiple comparisons test (**A**–**C** and **E**–**G**). *P < 0.05, **P < 0.01, ***P < 0.001, ****P < 0.0001. Scale bar: 50 μm. Ve, vehicle.

We then explored mechanisms behind *Pad2^–/–^* reduction in mortality and sepsis-induced organ injury in mice. Since PAD4 is a well-known key enzyme for NET formation, leading to vascular and lung injury during sepsis, we hypothesized that PAD2 also regulates NETosis. Since double-stranded DNA (dsDNA) is an important marker of NETs, we measured the concentrations of dsDNA in serum and peritoneal lavage fluid from CLP mice. Although the neutrophil numbers were not much changed in the WT and *Pad2^–/–^* mice after CLP (Supplemental Figure 4), the dsDNA concentrations in the samples of WT mice were significantly higher than those in the *Pad2^–/–^* mice (Figure 5, A and B).

To confirm the relationship between decreased NET formation and *Pad2^–/–^*, we next performed in vitro studies using neutrophils. We stimulated neutrophils with 2 different agonists involved in NETosis pathways: phorbol myristate acetate (PMA) and Ca^2+^ ionophore (A23187). PMA is known to activate the NADPH oxidase–dependent (NOX-dependent) pathway (17–19), and A23187 is known to stimulate the NOX-independent pathway (20). To measure the change of NET release in WT and *Pad2^–/–^* neutrophils, we used an assay with Sytox Green, the commonly used extracellular DNA dye. Compared with the WT group, the release of NETs was decreased in *Pad2^–/–^* neutrophils in response to both PMA and A23187, but the kinetics of release were not changed in *Pad2^–/–^* neutrophils (Figure 5C). To confirm this result, immunofluorescence staining and myeloperoxidase-DNA (MPO-DNA) assays were further conducted. The NETosis was reduced in *Pad2^–/–^* neutrophils in response to both activators after treatment for 3.5 hours (Figure 5, D, E, and G; and Supplemental Figure 5). It is well known that CitH3 is a marker of NETosis. Thus, we also examined effect of *Pad2* deficiency on CitH3 production. We found that CitH3^+^ neutrophils were also decreased in *Pad2^–/–^* neutrophils after stimulation with both agonists (Figure 5F). Overall, our results indicate that *Pad2^–/–^* inhibits both NOX-dependent and -independent pathways.

### Pad2^–/–^ increases macrophage number and decreases activation of noncanonical pyroptosis after CLP-induced sepsis.

**Figure 6:**
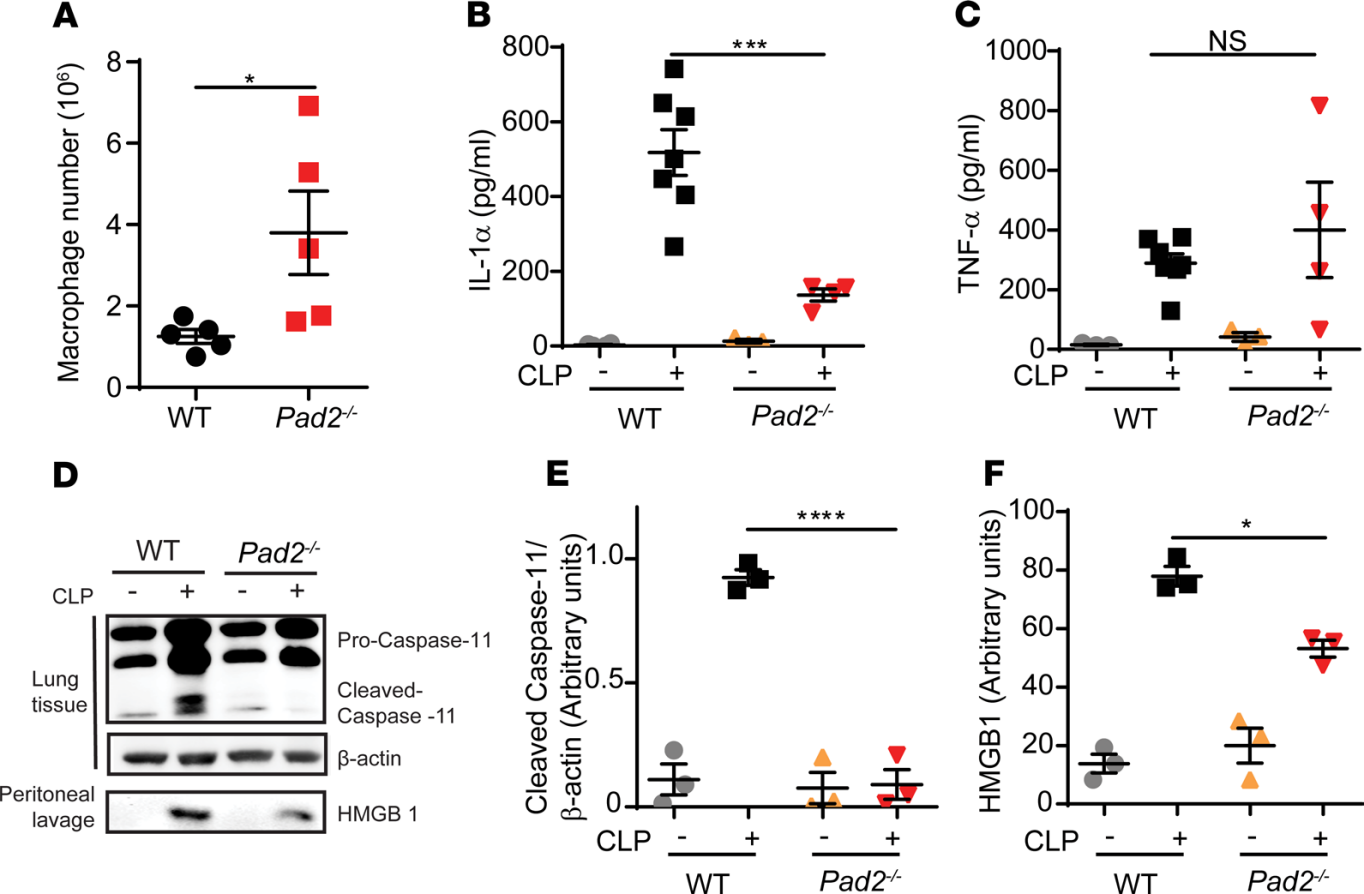
Absence of the *Pad2* gene inhibits Caspase-11–dependent noncanonical pyroptosis in a CLP murine model of sepsis. WT and *Pad2^–/–^* mice were subjected to CLP. (**A**) Macrophage number in peritoneal cavity of WT and *Pad2^–/–^* mice after 24 hours (*n* = 5/group). (**B** and **C**) Concentrations of IL-1α (**B**) and TNF-α (**C**) in peritoneal lavage of WT and *Pad2^–/–^* mice after 24 hours (*n* = 3–7/group). (**D**–**F**) Representative Western blot images and densitometry quantification of Caspase-11 activation in lung tissues (**D** and **E**) and HMGB1 concentration in peritoneal lavage of WT and *Pad2^–/–^* mice (**D** and **F**) after 24 hours. Western blot images are representative of at least 3 independent experiments. Data are expressed as mean ± SEM. Data with 2 groups were analyzed using unpaired 2-tailed Student’s *t* test (**A**). Data with 3 or more groups were analyzed by 2-way ANOVA with Bonferroni’s multiple comparisons test (**B**, **C**, **E**, and **F**). *P < 0.05, ***P < 0.001, ****P < 0.0001.

Since macrophages display important antimicrobial and proinflammatory properties during sepsis (21), we next determined whether PAD2 impacts macrophage activity in sepsis. First, we used flow cytometry to detect the macrophage number in the abdominal cavity at 24 hours following CLP. We found that the numbers of live macrophages were significantly elevated in *Pad2^–/–^* mice (Figure 6A).

Recent findings suggest that Caspase-11–induced macrophage death (noncanonical pyroptosis) is critical for CLP-induced sepsis (22). Therefore, we investigated whether an increase in the number of live macrophages in *Pad2^–/–^* mice was related to decreased noncanonical pyroptosis. Our study showed that Caspase-11 cleavage was decreased in lung tissue from *Pad2^–/–^* mice after CLP (Figure 6, D and E). In addition, the peritoneal levels of IL-1α (Figure 6B) and high-mobility group protein 1 (HMGB-1) (Figure 6, D and F), markers involved in the noncanonical pyroptosis pathway, were reduced in *Pad2^–/–^* compared with the WT mice (22, 23). In contrast, levels of TNF-α, a nonspecific proinflammatory cytokine for the noncanonical pyroptosis pathway (Figure 6C), was found to be no different in peritoneal lavage from *Pad2^–/–^* and WT mice, suggesting a unique role of PAD2 in Caspase-11 activation. To further confirm the involvement of PAD2*-*mediated Caspase-11 activation in CLP-induced sepsis, we assessed whether *Caspase-11^–/–^* mice display altered bacterial load and macrophage numbers similar to that observed in *Pad2^–/–^* mice. Like *Pad2^–/–^* mice, the bacterial clearance in the peritoneal cavity, blood, and spleen of *Caspase-11^–/–^* mice (Supplemental Figure 6A) was significantly increased, along with an increase of macrophage numbers (Supplemental Figure 6B) compared with WT mice. Together, these results indicate that the loss of *Pad2* function results in a reduction of noncanonical pyroptosis, thus increasing viable macrophage numbers that may contribute to the observed decrease in bacterial load and inflammation after CLP in *Pad2^–/–^* versus WT mice.

### Pad2^–/–^ inhibits noncanonical pyroptosis in BMDMs and the LPS-endotoxic model.

**Figure 7:**
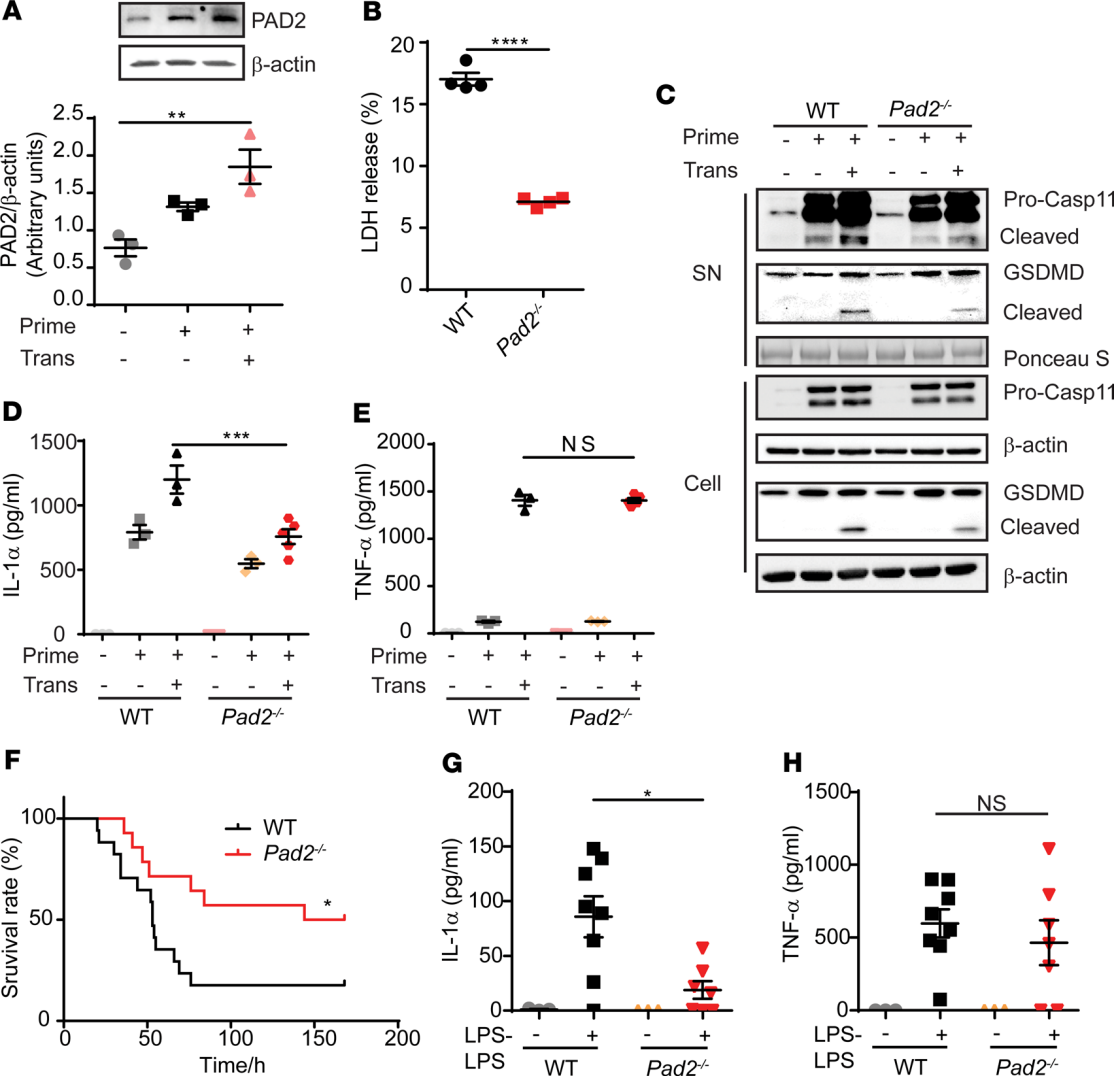
Absence of the *Pad2* gene inhibits Caspase-11–dependent noncanonical pyroptosis in BM-derived macrophages (BMDMs) and LPS-endotoxic model. (**A**) Representative Western blot images and densitometry quantification show expression of PAD2 in WT mouse BMDMs after noncanonical pyroptosis treatment. (**B**) LDH release in the supernatant of BMDMs after noncanonical pyroptosis treatment. (**C**) Representative Western blot images show Caspase-11 and GSDMD activation in the supernatant and cell lysate of BMDMs with or without noncanonical pyroptosis treatment. (**D** and **E**) Concentrations of IL-1α (**D**) and TNF-α (**E**) in the supernatant of BMDMs with or without noncanonical pyroptosis treatment (*n* = 3/group). (**F**) Kaplan-Meier survival curve of WT and *Pad2^–/–^* mouse in LPS-endotoxic mouse model (*n* = 14–17/group). (**G** and **H**) Concentrations of IL-1α (**G**) and TNF-α (**H**) in the serum of WT and *Pad2^–/–^* mice with or without LPS challenge (*n* = 3, 8, 3, and 7 for each group, respectively). Results in **A**–**E** were representative of at least 3 independent experiments. Data are expressed as mean ± SEM. Data with 2 groups were analyzed using unpaired 2-tailed Student’s *t* test (**B**). Three or more groups were analyzed by 1-way (**A**) or 2-way (**D**, **E**, **G**, and **H**) ANOVA with Bonferroni’s multiple comparisons test. Survival curve were analyzed by log-rank test. *P < 0.05, **P < 0.01, ***P < 0.001, ****P < 0.0001. SN, supernatant.

To further verify that *Pad2^–/–^* inhibits noncanonical pyroptosis, BMDMs from WT and *Pad2^–/–^* mice were primed and then transfected with LPS using a DNA transfection reagent, the standard method to active noncanonical pyroptosis pathway in vitro (22). We first assessed whether PAD2 expression is changed during noncanonical pyroptosis. We found that PAD2 protein levels were upregulated after transfection with LPS, while only moderately increased in the LPS-primed group (Figure 7A). In line with in vivo studies, *Pad2^–/–^* decreased Caspase-11–activated noncanonical pyroptosis, which was manifested by reduced LDH release, Caspase-11 cleavage, gasdermin D (GSDMD) cleavage, and IL-1α secretion in the *Pad2^–/–^* group (Figure 7, B–D). There was no difference in TNF-α levels between WT and *Pad2^–/–^* groups (Figure 7E).

A small dose followed by a higher concentration of LPS has been used to mimic noncanonical pyroptosis in vivo (24). Similarly, *Pad2^–/–^* mice had significantly decreased IL-1α release and lethality in the LPS model, but there was no change in TNF-α release (Figure 7, F–H).

Multiple genes in the noncanonical pyroptosis pathway were also analyzed in the BMDMs of *Pad2^–/–^* mice transfected with LPS in vitro. We found that none of these genes was significantly changed compared with the WT group (Supplemental Figure 7). These results imply that *Pad2^–/–^* inhibits noncanonical pyroptosis but that this inhibition does not occur through gene regulation.

### Pad4^–/–^ does not improve survival in the CLP model but enhances noncanonical pyroptosis activation in vitro.

As previous studies have shown, *Pad4* deficiency may not improve outcomes in polymicrobial sepsis (11). We validated this finding in *Pad4^–/–^* mice (Supplemental Figure 8) and mice treated with PAD4 selective inhibitor GSK484 (Figure 2B). There was no significant difference in survival between WT and *Pad4^–/–^* mice, or between the mice treated with and without GSK484.

PAD4 is also well known for its function in NET formation. Therefore, we sought to evaluate whether the different roles of PAD2 and PAD4 in sepsis could be attributable to differential activation of noncanonical pyroptosis. Interestingly, we found that *Pad4^–/–^* BMDMs underwent the enhanced noncanonical pyroptosis in an in vitro model, as reflected by increased LDH release, Caspase-11 cleavage, GSDMD cleavage, and IL-1α release compared with BMDMs from WT mice; TNF-α levels were not different between WT and *Pad4^–/–^* groups (Supplemental Figure 9). Our findings revealed that inhibition of PAD4 can enhance Caspase-11–dependent pyroptosis. Taken together, our studies suggest that regulation of Caspase-11 activation by PAD2/PAD4 contributes to macrophage noncanonical pyroptosis and animal survival during sepsis.

## Discussion

Increasing evidence has highlighted the importance of PAD2 in various immune and inflammatory disorders, including rheumatoid arthritis and cancers (25–27). However, little is known about the functions of PAD2 in sepsis. In this study, we demonstrated that PAD2 is elevated in serum and BALF of patients with sepsis, as well as in the blood and lung tissues of mice subjected to CLP. We found that *Pad2* deficiency decreases NETosis and macrophage Caspase-11–dependent pyroptosis, thereby increasing macrophage numbers, reducing bacterial loads and inflammation, and ultimately increasing survival and organ functions following the onset of sepsis.

### Elevated PAD2 correlates with severity of sepsis and organ injury in human patients and mice.

Circulating PAD2 has been reported in inflammatory states of coronary artery bypass surgery (28) and arthritis (29). We demonstrate in the current study that PAD2 is also elevated in the serum of septic patients and BALF from patients with sepsis-induced ARDS. It is consistent with the findings from our group that the *Pad2* gene was upregulated in alveolar macrophages and buffy coat cells from patients with ARDS (30). In our study, the increased concentration of PAD2 is associated with high levels of lactate or PCT, suggesting that PAD2 is correlated with severity of sepsis and organ injury in human patients (31). In animals, PAD2 protein is increased in the LPS-treated BMDM ex vivo and is elevated in serum and lung tissues in the sepsis mouse model. It is still not completely clear how and why PAD2 levels are upregulated during sepsis. Recent studies revealed that inflammation and bacterial infection induce expression of PAD2 in macrophages and other immune cells (32–34). Upon induction, PAD2 can be actively released or passively leaked (8, 28) into body fluids, which may cause altered host response to sepsis. Moreover, extracellular PAD2 could citrullinate local or circulating proteins (e.g., HMGB1) for further signal transduction (35, 36). Our findings highlight a potential role of PAD2 in NETosis and macrophage pyroptosis in the pathogenesis of sepsis.

### Bacterial clearance regulated by Pad2^–/–^ is not related to NET inhibition.

NETosis was first reported by Brinkmann et al. in 2004 (3). They found that neutrophils release a NET-like structure that can trap and kill bacteria during infections. Since then, many researchers reported this “trap” and “bactericidal” effect of NETosis (37). However, the role of NETosis in bacterial killing during infection has been questioned in recent years. Instead, more evidence has emerged to demonstrate that NETs cause more harm than good during infectious processes. Menegazzi et al. reported that bacteria released from NETs by DNase remain viable after long-time NET exposure (38). Similar results have demonstrated that bacteria bound to PMA-induced NETs also remain viable (39). Furthermore, an in vivo study indicates a dispensable bactericidal effect of NETs (40). PAD4 is a pivotal enzyme for NETosis, and its deficiency does not alter mortality nor bacterial load in CLP-induced sepsis (11). In our present study, *Pad2* genetic deficiency dramatically decreases bacterial load in blood while inhibiting NETosis, suggesting that NET formation does not necessarily correlate with bacterial killing.

### Pad2 deficiency may attenuate NET-induced inflammation.

Inhibiting NETosis or NET components during infection appears to be beneficial to the host (41–44). During NETosis, components from damage-associated molecular patterns (DAMPs) such as dsDNA, neutrophil elastase (NE), and histones released from neutrophils induce production of proinflammatory cytokines, increase vascular permeability, and cause organ damage (e.g., ALI) (45).

Several previous studies reported that PAD2 is not required for NET formation (46, 47). However, while they used low concentrations of PMA, ionomycin, or LPS to induce small percentage of NET formation, we used higher concentrations of PMA and A23187 to induce an abundance of NET formation on the WT group. Combined with several ex vivo quantitative research tools (48–50), we found that *Pad2* deficiency can reduce the speed and quantity of NET formation, although it does not fully abolish NETosis. Nevertheless, by decreasing NET formation, *Pad2^–/–^* can decrease the downstream proinflammatory effect, thus mitigating vascular permeability and organ damage.

### Pad2^–/–^-induced bacterial clearance may be associated with enhanced phagocytosis and decreased noncanonical pyroptosis in macrophages.

Macrophages are of particular interest to our study, since we found that *Pad2^–/–^* enhances phagocytosis of macrophages, instead of neutrophils. In addition, the increased macrophage numbers in the peritoneal lavage of *Pad2*^–/–^ mice after CLP further indicates a significant change in the state of macrophages. Pyroptosis is an inflammatory form of cell death that occurs mainly in macrophages. This form of cell death can be induced through 2 different signaling pathways: Caspase-1–dependent pyroptosis and Caspase-11–dependent noncanonical pyroptosis. While Caspase-1–mediated pyroptosis plays an important role in chronic autoimmune and other inflammatory diseases, Caspase-11–dependent noncanonical pyroptosis exacerbates pathologies in mouse models of sepsis (22, 24, 51–53). In this pathway, cytosolic LPS from Gram-negative bacterium activates Caspase-11 (Caspase-4/5 in humans) to cleave GSDMD, which then triggers cell membrane pore formation and cell death of macrophages (54, 55). Meanwhile, Caspase-11 mediated extracellular release of IL-1α and HMGB1 directly and induces secretion of mature IL-1β and IL-18 indirectly through engagement of the canonical Nlrp3 inflammasome (22, 23). IL-1α is a typical marker specific in Caspase-11–dependent pyroptosis (22). HMGB1 serves as both a marker and mediator in the Caspase-11–dependent pyroptosis pathway (23, 24). It was reported that PAD2 regulates the activation of Caspase-1 inflammasome (56), but the effects of PAD2 on Caspase-11–dependent pyroptosis has not been studied. In our current study, we found that PAD2 activates Caspase-11–dependent pyroptosis. *Pad2* deficiency can significantly decrease Caspase-11 activation both in vitro and in vivo. Inhibition of Caspase-11 activation leads to less IL-1α and HMGB1 release in the peritoneal cavity (Figure 6), and it reduces abdominal inflammation during sepsis. Moreover, reduced Caspase-11 activation in the absence of *Pad2* protects macrophages from pyroptosis-induced cell death during infection. Such a protective effect was also observed in separate experiments using *Caspase-11^–/–^* mice as a positive control.

PAD2 is also an important gene regulator that can translocate into the nucleus to convert histone H3 to CitH3 and alter gene expression (57, 58). However, our study showed that PAD2 regulated noncanonical pyroptosis in a manner that is not necessarily through gene regulation. Therefore, protein citrullination could be a potential mechanism to explain this effect. For example, HMGB1 has been shown to be one of the most important substrates of PAD2 (36). Given the important role of posttranslational modified HMGB1 (e.g., acetylation) in both sepsis and the Caspase-11–dependent pyroptosis pathway (24), it is possible that citrullinated HMGB1 by PAD2 could increase its release and further activate Caspase-11.

### Pad4 deficiency does not alter survival but enhances Caspase-11–dependent pyroptosis.

PAD2 and PAD4 are very important immune regulators. These proteins may exert synergistic effects on some pathways (such as NLRP3 inflammasome activation) (56) while having distinct effects on other pathways (59). Based on our result (Supplemental Figure 8) and findings of others (11), *Pad4-*KO mice were not protected in experimental mouse sepsis. However, targeting specific components of NETs can improve survival of sepsis (41, 43, 44, 60). Therefore, PAD4 may also be involved in pathways during sepsis other than NETosis. *Pad2^–/–^* decreases macrophage pyroptosis; therefore, we suspected *Pad4* deficiency might also alter the process of pyroptosis. Interestingly, we found that *Pad4^–/–^* can increase Caspase-11 activation, GSDMD cleavage, and IL-1α and LDH release in the BMDM, a typical phenomenon of noncanonical pyroptosis (Supplemental Figure 9). Our study indicates that PAD2 and PAD4 play opposite roles in Caspase-11–dependent pyroptosis, which potentially explains the different functions of PAD2 and PAD4 in sepsis.

In conclusion, to our knowledge, this is the first mechanism study of *Pad2* gene on sepsis. We found that PAD2 is elevated in both human septic patients and in mouse sepsis, and *Pad2^–/–^* mice or treatment of mice with a PAD2 specific inhibitor can increase the survival and improve outcomes in sepsis. Furthermore, we demonstrated that *Pad2^–/–^* inhibits NET formation and Caspase-11–dependent pyroptosis. Given the lack of effective therapeutic targets for sepsis, our study provides insights into the molecular mechanism of PAD2 in pathogenesis and treatment of sepsis.

## Methods

### Human subjects.

Serum samples of septic patients were collected from the emergency department or intensive care unit of University of Michigan. Patients with the diagnosis of sepsis were consistent with consensus definitions for severe sepsis or septic shock at the time during collection (61). The sample preparation and patient information have been described previously (62).

BALF of sepsis-induced ARDS patients was collected from the ALI Specialized Center of Clinically Oriented Research (SCCOR) as a part of randomized trial of granulocyte-macrophage CSF administration (ClinicalTrials.gov NCT00201409) conducted at the University of Michigan (63). The sample preparation and patient information have been described previously (64).

Healthy volunteers were asymptomatic, ambulatory nonsmokers under 60 years of age, who had no known chronic medical conditions and were taking no medications.

### Mice.

All experiments were performed using 8- to 12-week-old male mice unless otherwise mentioned. *Pad2-*KO mice generated on FVB/NJ background were provided by Scott Coonrod (Cornell University, Ithaca, New York, USA). Caspase-11^–/–^ mice generated on a C57BL/6J background were provided by Gabriel Nunez (University of Michigan). FVB/NJ and C57BL/6J WT mice were purchased from the Jackson Laboratory and used as controls for *Pad2^–/–^* mice and *Caspase-11^–/–^* mice in all studies. *Pad4^–/–^* mice were generated on C57BL/6J × SJL mixed background in our laboratory and were backcrossed into C57BL/6J for 7 generations before experiments. Littermate WT mice were used as controls in the experiment. All animal experiments were performed with an approval from the University of Michigan (PRO00008861).

### Development of PAD4-KO mice.

*PAD4*-KO mice were generated using CRISPR/Cas9 technology. Briefly, A premature stop codon followed by a unique restriction motif (for genotyping) was inserted near the start codon of *PAD4* gene, which would result in a halted translation following residue 10. The deleted 65 bp genomic sequence is as follows: 5′-CGGACATCCAGCGGGGTCGCTGTGCCCACCACACACACGGCGTGAGTGGGCTGCTCGGGGGCCAC-3′. Then, a 14 bp sequence was inserted at the same site, which is 5′-AATTCTCATTATCA-3′. Afterward, these generated *PAD4*-KO mice were mated with WT mice of C57BL/6J background to establish germline transmission. Resultant heterozygotes were used to generate homozygotes of *PAD4*-KO mice.

### ELISA.

Levels of PAD2 were measured using a commercial ELISA kit (PAD2 [human], Cayman Chemical, 501450; PAD2 [mouse], Aviva Systems Biology, OKCA02444). PCT concentrations were detected with a commercial ELISA kit (R&D Systems Inc., DY8350-05). All measurements were carried out in accordance with the industrial instruction. Concentrations of IL-1α, TNF-α, MIP-2, IL-6, and IP-10 were measured by the ELISA core of University of Michigan using the core-developed sandwich ELISA.

### CLP polymicrobial sepsis model.

Sepsis was induced by CLP. Briefly, the peritoneal cavity was opened under inhaled isoflurane anesthesia. The cecum was eviscerated, ligated below the ileocecal valve using a 5-0 silk suture at 75% from the tip, and punctured through with a 21-gauge needle. Sham-operated animals were handled in the similar manner, except the cecum was not ligated and punctured. For the survival study, mice were monitored for 7 days. For mechanistic studies, mice were sacrificed at their respective time point. For inhibitor analysis, PAD4 inhibitor GSK484 (40 mg/kg) (Cayman Chemical, 17488), PAD2 inhibitor AFM32a (20 mg/kg), which was synthesized as previously described (65), or DMSO alone was i.p. injected 1 hour after CLP.

### Bacterial burden determination.

Mice were euthanized 24 hours after CLP, and the peritoneal cavity was washed with 2 mL sterile PBS. Blood was collected through cardiac puncture, and the spleen was harvested and then homogenized in 1 mL sterile PBS (Gibco, Thermo Fisher Scientific). The blood, peritoneal lavage fluid, and spleen homogenate were then serially diluted in sterile PBS. The resulting diluent (100 μL) was plated onto tryptic soy agar plates and incubated at 37°C for 16 hours. Plates with colony numbers among 30–300 were counted. Results were expressed as CFU per milliliter blood, peritoneal lavage fluid, and spleen homogenate.

### Isolation of peritoneal macrophage.

Peritoneal macrophages were harvested in the mouse model of thioglycollate-induced sterile peritonitis (66). Briefly, 1.5 mL of 4% Brewer thioglycollate was peritoneally injected into mice. The animals were euthanized 3 days later, and the peritoneal cavity was flushed with 10 mL of cold PBS. Peritoneal macrophages were further collected after centrifugation (4°C, 400*g*, 10 minutes).

### Phagocytosis assay.

The phagocytotic ability of macrophages and neutrophils was evaluated using pHrodo Red *E. coli* BioParticles (Thermo Fisher Scientific, P35361). In brief, 2 × 10^5^ or 3 × 10^5^ cells per well were plated in a blank 96-well plate, and pHrodo Red *E. coli* BioParticles were suspended in living imaging solution at 1 mg/mL and sonicated to make it homogeneously dispersed. The macrophages and neutrophils were then coincubated with a bioparticle suspension for 1–1.5 hours at 37°C. Quantitative results were read using fluorescence plate reader (Promega, E8032). Mean values are RFU, calculated from 3–4 wells per group, and subtracting the baseline fluorescence from wells containing pHrodo *E*. *coli* BioParticles but no cells (means ± SEM). Representative images were obtained using a fluorescence microscope (Olympus, DP70). Six microscopic fields were randomly chosen from each group.

### Vascular permeability assay.

Microvascular permeability was evaluated by the Evans Blue (EB) assay. In brief, 1% EB in normal saline (4 mL/kg) was injected to mice through tail veins 18 hours after CLP. Mice were sacrificed after 30 minutes and perfused with 25 mL PBS. Tissues were harvested, and 50–100 mg tissue of each organ was homogenized with 500 μL dimethylformamide (DMF) and then incubated at 55°C for 27 hours. Tissue lysates were centrifuged at 4°C and 7000*g* for 10 minutes, and the supernatant was collected for optical density (OD) measurement at A620. The accurate EB weight for each organ was further quantified according to a standard curve made by serial dilution of known concentration of EB solution.

### Lung injury analysis and IHC.

Mice were euthanized 24 hours after CLP. For inflammatory cytokine detection, the lungs were flushed with 3 mL sterile HBSS to get the BALF samples. Inflammatory cytokines in the BALF were detected using ELISA. For histological analysis of ALI, the lung tissues were embedded in paraffin and sliced into 5 μm sections. H&E staining was performed for histology detection. The ALI scoring was conducted by a pathologist blinded to the experiment groups. ALI was classified into 6 categories based on the parameters of (a) neutrophils, (b) septal hemorrhage and congestion, (c) septal mononuclear cell/lymphocyte infiltration, (d) alveolar hemorrhage, (e) alveolar macrophages, and (f) alveolar edema. The severity of each category was graded from 0 (minimal) to 3 (maximal), and the total score was calculated by adding the scores in each of these categories.

Immunohistochemical staining of macrophages (F4/80^+^) and neutrophils (MPO^+^) were performed using Immunohistochemistry Application Solutions Kit (Rabbit) (Cell Signaling Technology, 13079) according to the manufacturer’s instruction. Anti-F4/80 (Cell Signaling Technology, 70076S, 1:1000) and anti-MPO (Abcam, ab9535, 1:400) were used as primary antibodies. Negative controls for immunohistochemical analysis were the sections incubated with antibody dilution buffer without the primary antibodies, which showed no positive staining. The positively stained cells were counted from 3 different areas under high-power field (×400), and the final positive cell numbers of each slides resulted from the average of the 3 areas.

### Serum levels of dsDNA measurement.

The PicoGreen assay kit (Invitrogen, Thermo Fisher Scientific, P11495) was used to detect circulating dsDNA per manufacturer’s instructions. Briefly, the serum was incubated with the PicoGreen assay reagent for 5 minutes and then quantified with fluorescence plate reader.

### NETosis induction and detection.

Neutrophils used in this study were BM neutrophil isolated using EasySep Mouse Neutrophil Enrichment Kit (Stemcell Technologies, 19762). Cells were suspended with HBSS with calcium chloride/magnesium chloride and 2% FBS. NETosis was induced using Calcium Ionophore (A23187, 25 μM) or PMA (100 nM).

For the plate-reader assay, cells were seeded at 1 × 10^5^ cells per well in a blank 96-well plate in the presence of 5 μM Sytox Green. The fluorescence was measured by a fluorescence plate reader (Promega, E8032) at 1-hour time intervals for up to 8 hours after cell activation. For the NETosis index, a fluorescence readout obtained from cells lysed using 0.33% Triton-100 was considered as 100% DNA release, and a signal from the untreated group was considered as from spontaneous DNA release. The index was calculated as the percentage of total DNA released after subtracting the background signal (49).

For visualization of NETs, 8-well chamber slides were coated with poly-l-lysine and seeded with 1 × 10^5^ cells per well. After treatment with PMA or Calcium Ionophore for 3.5 hours, the cells were fixed and permeabilized with neutral-buffered formalin and methanol. Cells were blocked with 3% BSA prepared in PBS for 1 hour at room temperature and then incubated with primary antibodies overnight at 4°C and respective secondary antibodies for 1 hour at room temperature. DNA was stained with mounting medium containing DAPI. The primary antibodies used were CitH3 (4D5D6) (60) and MPO (Abcam, 9535). Imaging was obtained using a fluorescence microscope (Olympus, BX53). Six microscopic fields were randomly chosen from each group, and the percentage of CitH3^+^ neutrophils or those undergoing NETosis was determined by blinded manual counting.

### MPO-DNA assay.

To quantify the MPO-DNA complex in the cell supernatant, a capture ELISA based on MPO associated with DNA was performed (50). Cell supernatant was pretreated with limited DNase digestion. Anti-MPO antibody (Invitrogen, Thermo Fisher Scientific, PA5-16672, 1:1000) was coated overnight as a capture antibody. After blocking, the diluted cell supernatant was then added to the plate and incubated at room temperature for 3 hours. Peroxidase-labeled anti-DNA monoclonal antibody (Roche, Cell Death Detection ELISA, 11544675001) was used as the detection antibody. After incubating for 1.5 hours at room temperature, the peroxidase substrate (TMB) was added, and the reactions were then ended by stop solution. The final results were shown as the OD value at 450 nm measured by microplate reader (Molecular Devices, Spectra Max Plus).

### Flow cytometry.

Peritoneal immune cell numbers were measured using flow cytometry. Peritoneal cavities were flushed with cold PBS and then filtered through a 70 μm cell strainer to get rid of leaking feces. Next, total peritoneal cells were counted and stained with specific fluorochrome-conjugated monoclonal antibodies (all from BioLegend) (F4/80, 123115; CD45, 103137; Ly6G, 45-5931-80; CD11b, 101223). Dead cells were excluded according to 7-ADD staining (BD Pharmingen, 51-68981E). Fluorescence data were acquired on a LSRFortessa Flow Cytometer (BD Biosciences) and analyzed using FlowJo software (Tree Star Inc.).

### Blood neutrophil count.

Mouse blood neutrophils were counted using VETSCAN HM5 Hematology Analyzer according to the manufacturer’s instruction.

### Isolation of BMDMs.

BMDMs were generated using well-established protocols. In brief, BM cells were flushed out of mouse legs with 60 mL of complete medium of IMDM supplemented with 20% L929 cell medium, penicillin, streptomycin, 2-mercaptoethanol, glutamine, and 10% heat-inactivated fetal calf serum (Gibco, Thermo Fisher Scientific). The fresh completed medium (30 mL) was added at day 3. Adherent BMDMs were harvested at day 7 and then used for following experiments.

### Macrophage transfection.

BMDMs were plated in a 6-well plate at 1 × 10^6^ cells per well. Cells were first primed with 50 ng/mL LPS in Opti-MEM overnight. For LPS transfection, 1.5 μg of LPS were packed with 7.5 μL of DOTAP after 30 minutes of incubation at room temperature, and the mixture was diluted into 1 mL of Opti-MEM before adding to 6-well plates. IL-1α and TNF-α release in cell-free supernatant was measured by ELISA. Cell lysate was analyzed by Western blot with antibodies against Caspase-11, GSDMD, and β-actin.

### Lactate dehydrogenase (LDH) assay.

LDH released from pyroptotic cells was measured by a Cytotoxicity Detection LDH Kit (Roche, 11644793001). In brief, 10,000 BMDMs per well were seeded in a 96-well plate and treated with or without LPS transfection. For LDH release detection, 100 μL supernatant was collected from each well. After centrifugation (4°C, 350*g*, 5 minutes), 100 μL of the kit’s reaction mixture were added and coincubated for 30 minutes in the dark. The microplate reader (Molecular Devices, Spectra Max Plus) was used to measure the OD at 490 nm. The final LDH release was calculated according to the manufacturer’s protocol.

### Endotoxemia mouse model.

The endotoxemia mouse model was induced through 2 shot LPS; mice were first injected i.p. with a low dose of LPS (400 μg/kg) (MilliporeSigma, L2630) for 6 hours and then challenged with a high dose of LPS (4 mg/kg). For survival study, mice were monitored for 7 days. For serum cytokine detection, mice were sacrificed 4 hours after the second LPS challenge.

### Western blotting.

The antibodies were purchased from the following: anti-PAD2 (Proteintech, 12110-1-AP), anti–Caspase-11 (Abcam, 180673), anti–GSDMD (Abcam, 209845), and anti-HMGB1 (Abcam, ab18256). Cell and tissue lysates were prepared using radioimmunoprecipitation assay (RIPA buffer). Protein in cell supernatant were precipitated by deoxycholate-trichloroacetic acid method, and Ponceau S staining was used as loading control. For peritoneal lavage preparation, 30 μL peritoneal lavage with 6 μL 6× loading buffer were directly used as loading samples. Since there is no ideal loading control for peritoneal lavage, a loading volume of 30 μL sample was fixed in all groups.

### Quantitative PCR.

Total RNAs were extracted using RNeasy Mini Kit (QIAGEN, 74104) and were reverse-transcribed into cDNA (Applied Biosystems, Thermo Fisher Scientific, 4368814). Quantitative PCR was performed with iQ SYBR Green Supermix (Bio-Rad). Gene expression was normalized against the internal control GAPDH. The primer sequences are shown in Supplemental Table 1.

### Statistics.

Statistical analyses were performed using Prism software (GraphPad). Normality of human data pools was tested with the Pearson-D’Agostino normality test. As for nonnormality distribution, Kruskal-Wallis test with Dunnett’s multiple comparisons test were used to compare multiple groups. Mann-Whitney *U* test was used to compare 2 groups. Association of PAD2 with lactate and PCT were studied using a Pearson regression model. For other in vivo and in vitro studies, when the sample number was too small for normality detection, the 1-way or 2-way ANOVA with Bonferroni’s multiple comparisons test was used to compare multiple groups. An unpaired 2-sided *t* test was used to compare 2 groups. Survival curves were analyzed by log-rank test. A *P* value less than 0.05 was considered significant for all experiments.

### Study approval.

All animal studies were reviewed and approved by the University Committee on the Use and Care of Animals (University of Michigan; PRO00008861 and PRO00008239). Human studies used protocols approved by the University of Michigan IRB (HUM 00056630, IRB 2003-0430, and IRB 2003-0829). Written informed consent was obtained from all participants or their legal proxy for medical decision-making before study inclusion.

## Author contributions

YL, YT, HBA, and TJS contributed to the concept and design of the study. KAS and TJS provided the clinical data and samples. SM and PRT synthesized the PAD2 inhibitor compound. YT, SQ, ZW, QD, BP, JZ, BL, and XD contributed to the performance of the assays. YT performed all the statistical analysis and wrote the manuscript. YL, HBA, AMW, KAS, JM, PRT, and TJS contributed to the revision of manuscript. All authors read the final approved manuscript.

## Supplementary Material

Supplemental data

## Version 1 10/15/2020

Electronic publication

## References

[1] Singer M (2016). The Third International Consensus Definitions for Sepsis and Septic Shock (Sepsis-3). JAMA.

[2] Rudd KE (2020). Global, regional, and national sepsis incidence and mortality, 1990-2017: analysis for the Global Burden of Disease Study. Lancet.

[3] Brinkmann V (2004). Neutrophil extracellular traps kill bacteria. Science.

[4] Cao C, Yu M, Chai Y (2019). Pathological alteration and therapeutic implications of sepsis-induced immune cell apoptosis. Cell Death Dis.

[5] Delano MJ, Ward PA (2016). The immune system’s role in sepsis progression, resolution, and long-term outcome. Immunol Rev.

[6] Gao YL, Zhai JH, Chai YF (2018). Recent advances in the molecular mechanisms underlying pyroptosis in sepsis. Mediators Inflamm.

[7] Bicker KL, Thompson PR (2013). The protein arginine deiminases: structure, function, inhibition, and disease. Biopolymers.

[8] Zhou Y (2017). Spontaneous secretion of the citrullination enzyme PAD2 and cell surface exposure of PAD4 by neutrophils. Front Immunol.

[9] Vossenaar ER (2004). Expression and activity of citrullinating peptidylarginine deiminase enzymes in monocytes and macrophages. Ann Rheum Dis.

[10] Lewis HD (2015). Inhibition of PAD4 activity is sufficient to disrupt mouse and human NET formation. Nat Chem Biol.

[11] Martinod K (2015). PAD4-deficiency does not affect bacteremia in polymicrobial sepsis and ameliorates endotoxemic shock. Blood.

[12] Zhao T (2016). Protective effect of Cl-amidine against CLP-induced lethal septic shock in mice. Sci Rep.

[13] Witalison EE, Thompson PR, Hofseth LJ (2015). Protein arginine deiminases and associated citrullination: physiological functions and diseases associated with dysregulation. Curr Drug Targets.

[14] Zhou J (2020). Peptidylarginine deiminase 2 knockout improves survival in hemorrhagic shock. Shock.

[15] Opal SM, van der Poll T (2015). Endothelial barrier dysfunction in septic shock. J Intern Med.

[16] Cohen J (2002). The immunopathogenesis of sepsis. Nature.

[17] Hakkim A (2011). Activation of the Raf-MEK-ERK pathway is required for neutrophil extracellular trap formation. Nat Chem Biol.

[18] Douda DN, Yip L, Khan MA, Grasemann H, Palaniyar N (2014). Akt is essential to induce NADPH-dependent NETosis and to switch the neutrophil death to apoptosis. Blood.

[19] Keshari RS, Verma A, Barthwal MK, Dikshit M (2013). Reactive oxygen species-induced activation of ERK and p38 MAPK mediates PMA-induced NETs release from human neutrophils. J Cell Biochem.

[20] Wang Y (2009). Histone hypercitrullination mediates chromatin decondensation and neutrophil extracellular trap formation. J Cell Biol.

[21] Cavaillon JM, Adib-Conquy M (2005). Monocytes/macrophages and sepsis. Crit Care Med.

[22] Kayagaki N (2011). Non-canonical inflammasome activation targets caspase-11. Nature.

[23] Lamkanfi M, Dixit VM (2014). Mechanisms and functions of inflammasomes. Cell.

[24] Deng M (2018). The endotoxin delivery protein HMGB1 mediates caspase-11-dependent lethality in sepsis. Immunity.

[25] Sun B (2019). Reciprocal regulation of Th2 and Th17 cells by PAD2-mediated citrullination. JCI Insight.

[26] Darrah E (2018). Autoantibodies to peptidylarginine deiminase 2 are associated with less severe disease in rheumatoid arthritis. Front Immunol.

[27] Beato M, Sharma P (2020). Peptidyl arginine deiminase 2 (PADI2)-mediated arginine citrullination modulates transcription in cancer. Int J Mol Sci.

[28] Vejlstrup A, Møller AM, Nielsen CH, Damgaard D (2019). Release of active peptidylarginine deiminase into the circulation during acute inflammation induced by coronary artery bypass surgery. J Inflamm Res.

[29] Damgaard D, Senolt L, Nielsen CH (2016). Increased levels of peptidylarginine deiminase 2 in synovial fluid from anti-CCP-positive rheumatoid arthritis patients: association with disease activity and inflammatory markers. Rheumatology (Oxford).

[30] Kovach MA (2015). Microarray analysis identifies IL-1 receptor type 2 as a novel candidate biomarker in patients with acute respiratory distress syndrome. Respir Res.

[31] Castelli GP, Pognani C, Meisner M, Stuani A, Bellomi D, Sgarbi L (2004). Procalcitonin and C-reactive protein during systemic inflammatory response syndrome, sepsis and organ dysfunction. Crit Care.

[32] Damgaard D, Friberg Bruun Nielsen M, Quisgaard Gaunsbaek M, Palarasah Y, Svane-Knudsen V, Nielsen CH (2015). Smoking is associated with increased levels of extracellular peptidylarginine deiminase 2 (PAD2) in the lungs. Clin Exp Rheumatol.

[33] Arandjelovic S, McKenney KR, Leming SS, Mowen KA (2012). ATP induces protein arginine deiminase 2-dependent citrullination in mast cells through the P2X7 purinergic receptor. J Immunol.

[34] Funayama R (2017). Protein-arginine deiminase 2 suppresses proliferation of colon cancer cells through protein citrullination. Cancer Sci.

[35] Cloutier N (2013). The exposure of autoantigens by microparticles underlies the formation of potent inflammatory components: the microparticle-associated immune complexes. EMBO Mol Med.

[36] Lewallen DM (2015). Chemical proteomic platform to identify citrullinated proteins. ACS Chem Biol.

[37] McDonald B, Urrutia R, Yipp BG, Jenne CN, Kubes P (2012). Intravascular neutrophil extracellular traps capture bacteria from the bloodstream during sepsis. Cell Host Microbe.

[38] Menegazzi R, Decleva E, Dri P (2012). Killing by neutrophil extracellular traps: fact or folklore?. Blood.

[39] Mohanty T (2015). A novel mechanism for NETosis provides antimicrobial defense at the oral mucosa. Blood.

[40] de Jong HK (2014). Neutrophil extracellular traps in the host defense against sepsis induced by Burkholderia pseudomallei (melioidosis). Intensive Care Med Exp.

[41] Luo L (2014). Proinflammatory role of neutrophil extracellular traps in abdominal sepsis. Am J Physiol Lung Cell Mol Physiol.

[42] Li Y (2014). Citrullinated histone H3: a novel target for the treatment of sepsis. Surgery.

[43] Xu J (2009). Extracellular histones are major mediators of death in sepsis. Nat Med.

[44] Kolaczkowska E (2015). Molecular mechanisms of NET formation and degradation revealed by intravital imaging in the liver vasculature. Nat Commun.

[45] Meegan JE, Yang X, Coleman DC, Jannaway M, Yuan SY (2017). Neutrophil-mediated vascular barrier injury: role of neutrophil extracellular traps. Microcirculation.

[46] Holmes CL, Shim D, Kernien J, Johnson CJ, Nett JE, Shelef MA (2019). Insight into neutrophil extracellular traps through systematic evaluation of citrullination and peptidylarginine deiminases. J Immunol Res.

[47] Bawadekar M (2017). Peptidylarginine deiminase 2 is required for tumor necrosis factor alpha-induced citrullination and arthritis, but not neutrophil extracellular trap formation. J Autoimmun.

[48] Chen KW (2018). Noncanonical inflammasome signaling elicits gasdermin D-dependent neutrophil extracellular traps. Sci Immunol.

[49] Douda DN, Khan MA, Grasemann H, Palaniyar N (2015). SK3 channel and mitochondrial ROS mediate NADPH oxidase-independent NETosis induced by calcium influx. Proc Natl Acad Sci U S A.

[50] Sil P, Yoo DG, Floyd M, Gingerich A, Rada B (2016). High throughput measurement of extracellular DNA release and quantitative NET formation in human neutrophils in vitro. J Vis Exp.

[51] Kayagaki N (2013). Noncanonical inflammasome activation by intracellular LPS independent of TLR4. Science.

[52] Hagar JA, Powell DA, Aachoui Y, Ernst RK, Miao EA (2013). Cytoplasmic LPS activates caspase-11: implications in TLR4-independent endotoxic shock. Science.

[53] Tang Y (2018). TRIF signaling is required for caspase-11-dependent immune responses and lethality in sepsis. Mol Med.

[54] Shi J (2014). Inflammatory caspases are innate immune receptors for intracellular LPS. Nature.

[55] Schroder K, Tschopp J (2010). The inflammasomes. Cell.

[56] Mishra N (2019). Cutting Edge: Protein arginine deiminase 2 and 4 regulate NLRP3 inflammasome-dependent IL-1β maturation and ASC speck formation in macrophages. J Immunol.

[57] Clancy KW (2017). Citrullination/methylation crosstalk on histone H3 regulates ER-target gene transcription. ACS Chem Biol.

[58] Cherrington BD, Zhang X, McElwee JL, Morency E, Anguish LJ, Coonrod SA (2012). Potential role for PAD2 in gene regulation in breast cancer cells. PLoS One.

[59] Liu Y (2018). Peptidylarginine deiminases 2 and 4 modulate innate and adaptive immune responses in TLR-7-dependent lupus. JCI Insight.

[60] Deng Q (2019). Citrullinated histone H3 as a therapeutic target for endotoxic shock in mice. Front Immunol.

[61] Bone RC, Sibbald WJ, Sprung CL. The ACCP-SCCM consensus conference on sepsis and organ failure. *Chest*. 1992;101(6):1481–1483 10.1378/chest.101.6.1481 1600757

[62] Stringer KA (2015). Whole blood reveals more metabolic detail of the human metabolome than serum as measured by 1H-NMR Spectroscopy: implications for sepsis metabolomics. Shock.

[63] Paine R (2012). A randomized trial of recombinant human granulocyte-macrophage colony stimulating factor for patients with acute lung injury. Crit Care Med.

[64] Kovach MA (2017). IL-36γ is a crucial proximal component of protective type-1-mediated lung mucosal immunity in Gram-positive and -negative bacterial pneumonia. Mucosal Immunol.

[65] Muth A (2017). Development of a selective inhibitor of protein arginine deiminase 2. J Med Chem.

[66] Zhang X, Goncalves R, Mosser DM (2008). The isolation and characterization of murine macrophages. Curr Protoc Immunol.

