# Supplementary material for: Peptidylarginine deiminase 2 has potential as both a biomarker and therapeutic target of sepsis
Source: JCI Insight. 2020 Oct 15;5(20):e138873. doi: 10.1172/jci.insight.138873 (PMC7605547; doi:10.1172/jci.insight.138873)
Supplement: Supplemental data [file jciinsight-5-138873-s031.pdf]

Supplemental data

Peptidylarginine Deiminase 2: Mechanistic insights into its role in sepsis

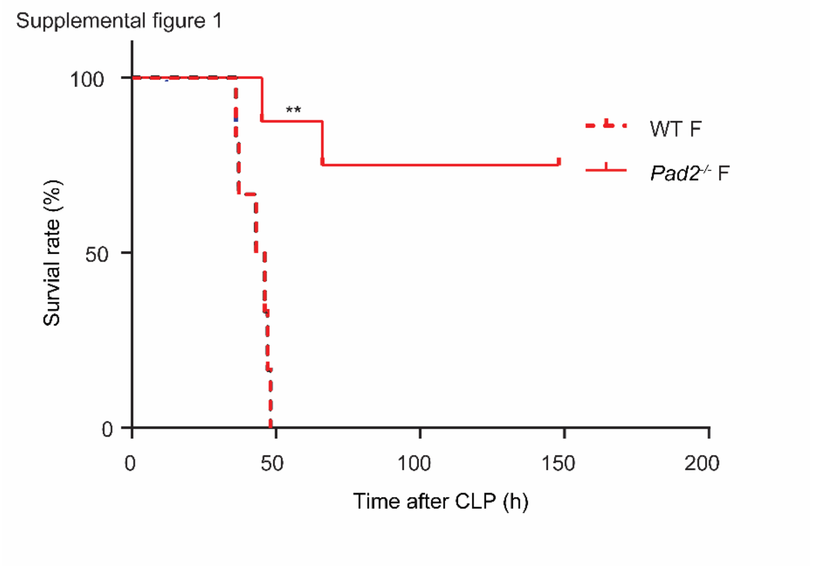

Supplemental figure 1: Absence of the *Pad2* gene improves survival in a female mouse model of sepsis. Kaplan-Meier survival curves of WT and *Pad2-/-* female mice subjected to **cecal-ligation and puncture (**CLP) (n=6 for female WT group and n=8 for female *Pad2-/-* group). Values are expressed as survival percentage. **p<0.01 by log-rank test; NS: not significant; F: female.

Supplemental figure 2

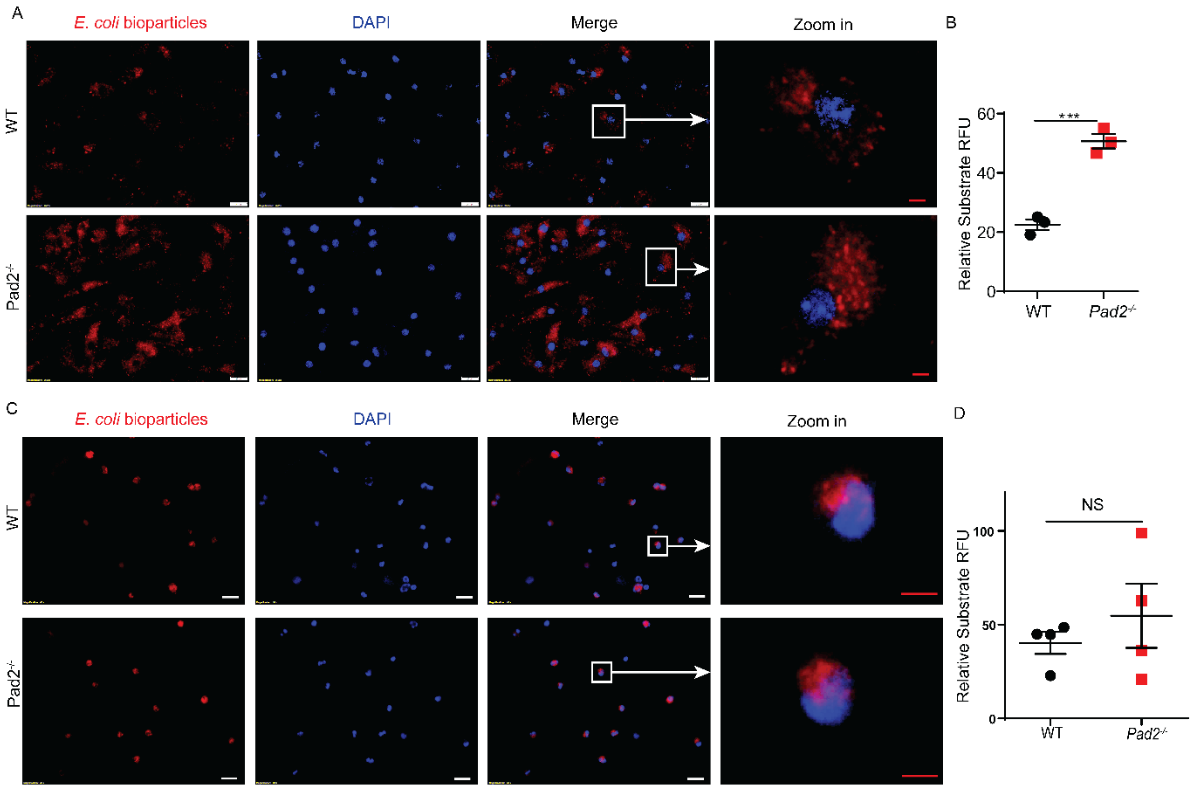

Supplemental figure 2: Absence of the *Pad2* gene enhances phagocytosis in bone marrow derived macrophages (BMDMs), not neutrophils. (**A**) Representative images show the engulfed *E. coli* bioparticles in BMDMs and (**B**) the fluorescence signal detected by a plate reader. (**C**) Representative images show the engulfed *E. coli* bioparticles in neutrophils and (**D**) the fluorescence signal detected by a plate reader. Values are relative fluorescence units (RFU), calculated from 3-4 wells per group. The results are representative of three independent experiments. Graphs are expressed as mean ± SEM. Data with two groups were analyzed using an unpaired Student's *t* test. NS: not significant. White scale bar: 20 µm, red scale bar: 5 µm.

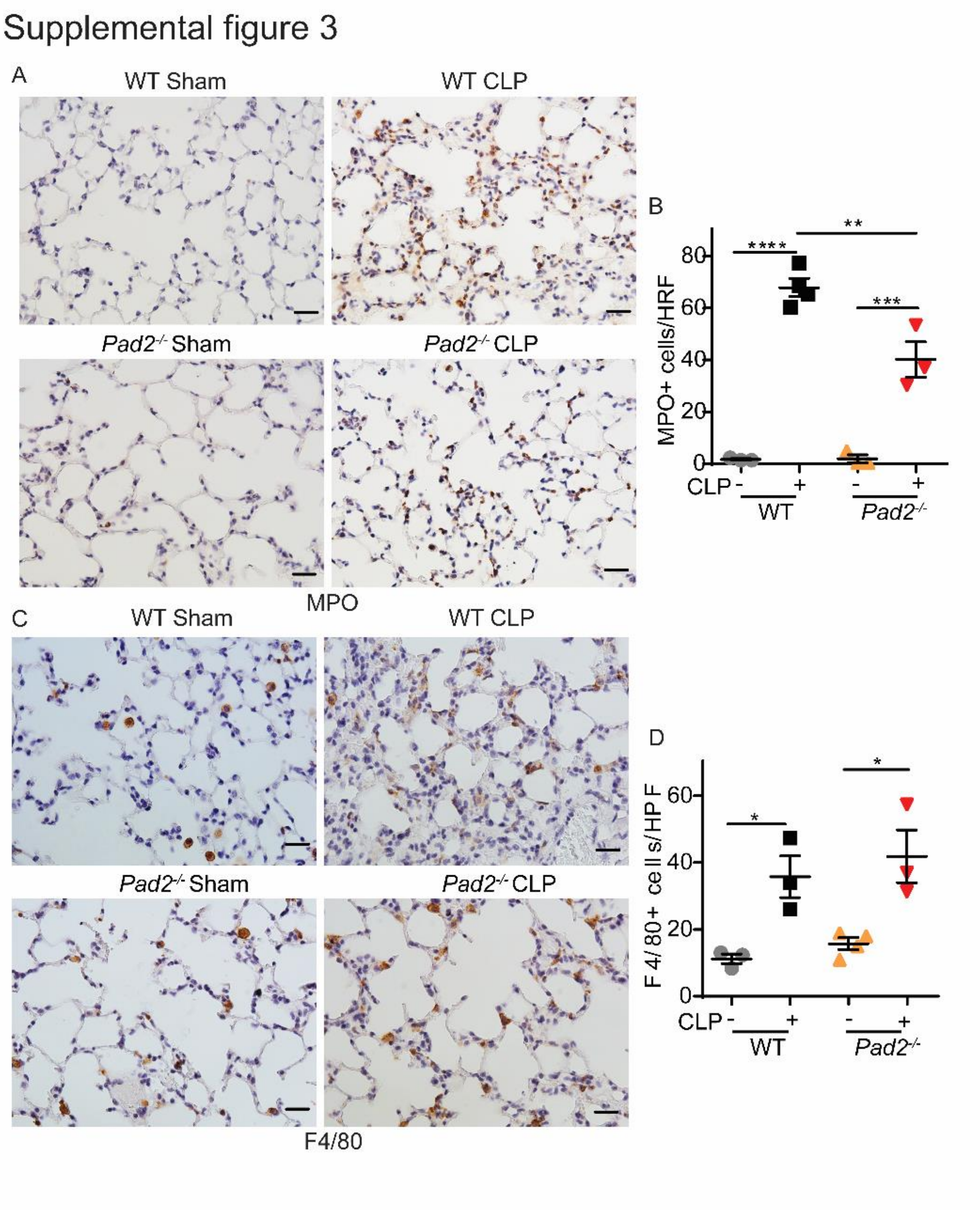

Supplemental figure 3: Absence of the *Pad2* gene ameliorates CLP-induced neutrophil infiltration in lung tissues. WT and *Pad2-/-* mice were subjected to CLP. (**A**) Immunohistochemical staining and (**B**) quantification of myeloperoxidase (MPO), a biomarker of neutrophil, in lung tissues. (**C**) Immunohistochemical staining and (**D**) quantification of F4/80, a biomarker of macrophage, in lung tissues. Graphs are expressed as mean ± SEM (n = 3-4/group). Data were analyzed using two-way ANOVA. *p<0.05, **p<0.01, ****p<0.0001. Scale bar: 20 μm.

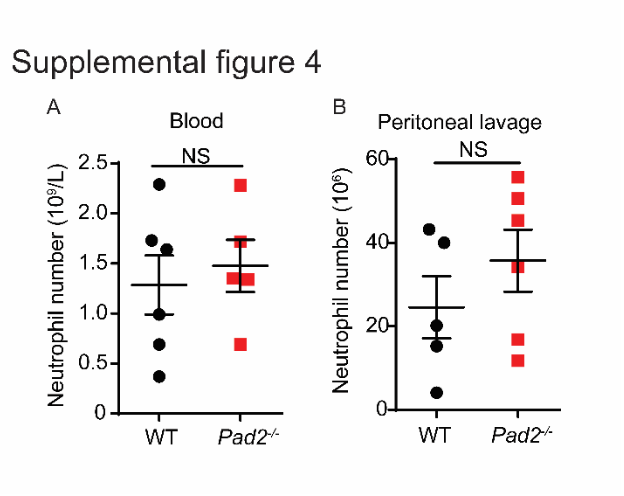

Supplemental figure 4: Absence of the *Pad2* gene does not affect neutrophil number in a cecal-ligation and puncture (CLP) murine model of sepsis. WT and *Pad2-/-* mice were subjected to CLP. Neutrophil numbers in (**A**) blood and (**B**) peritoneal cavity were measured 24 h after CLP. Graphs are expressed as mean ± SEM (n=5-6/group). Data were analyzed using an unpaired Student's *t* test. NS: not significant.

Supplemental figure 5

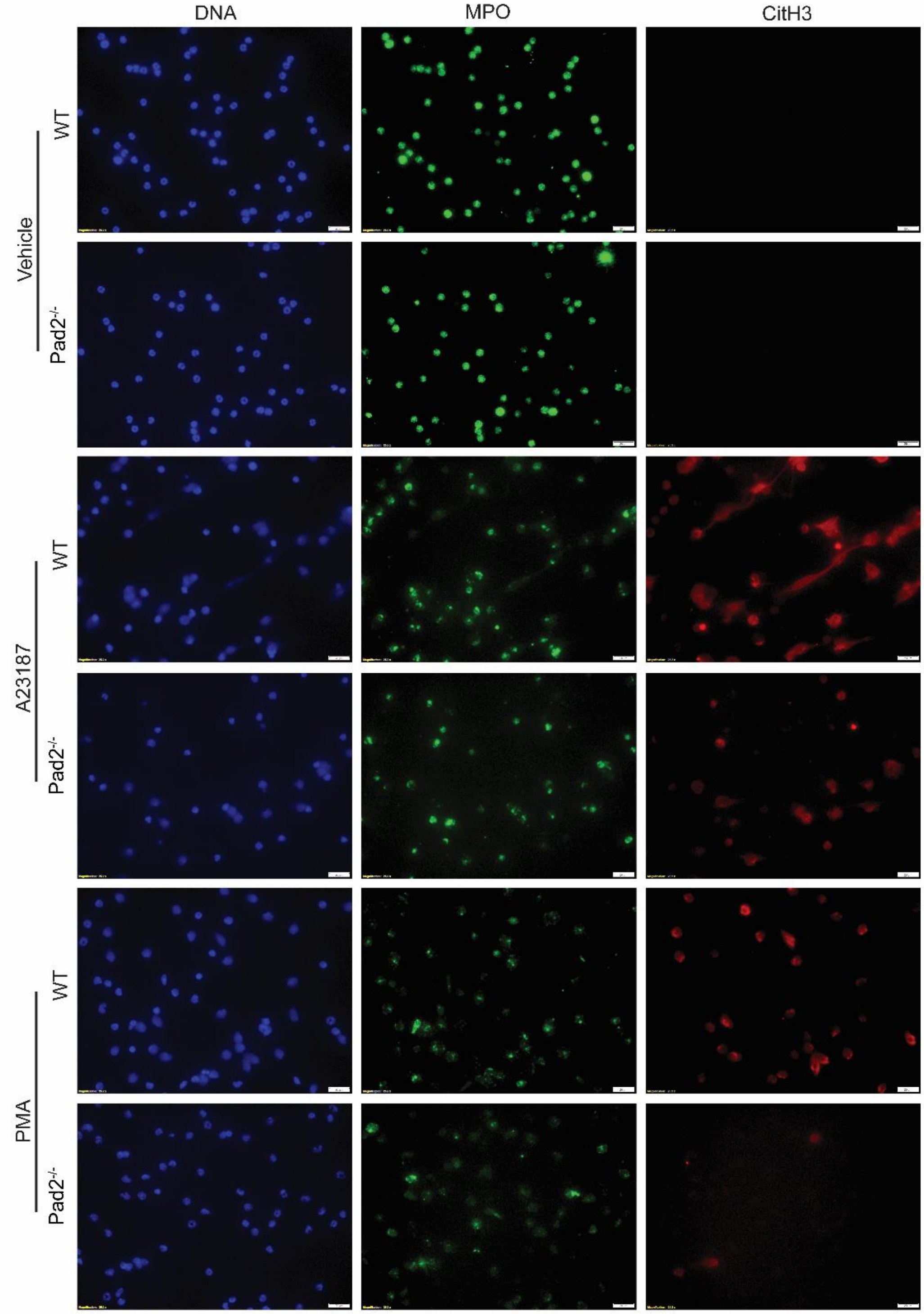

Supplemental figure 5: Single color staining images of NETs showed in Figure 5D. Scare bar: 20 μm.

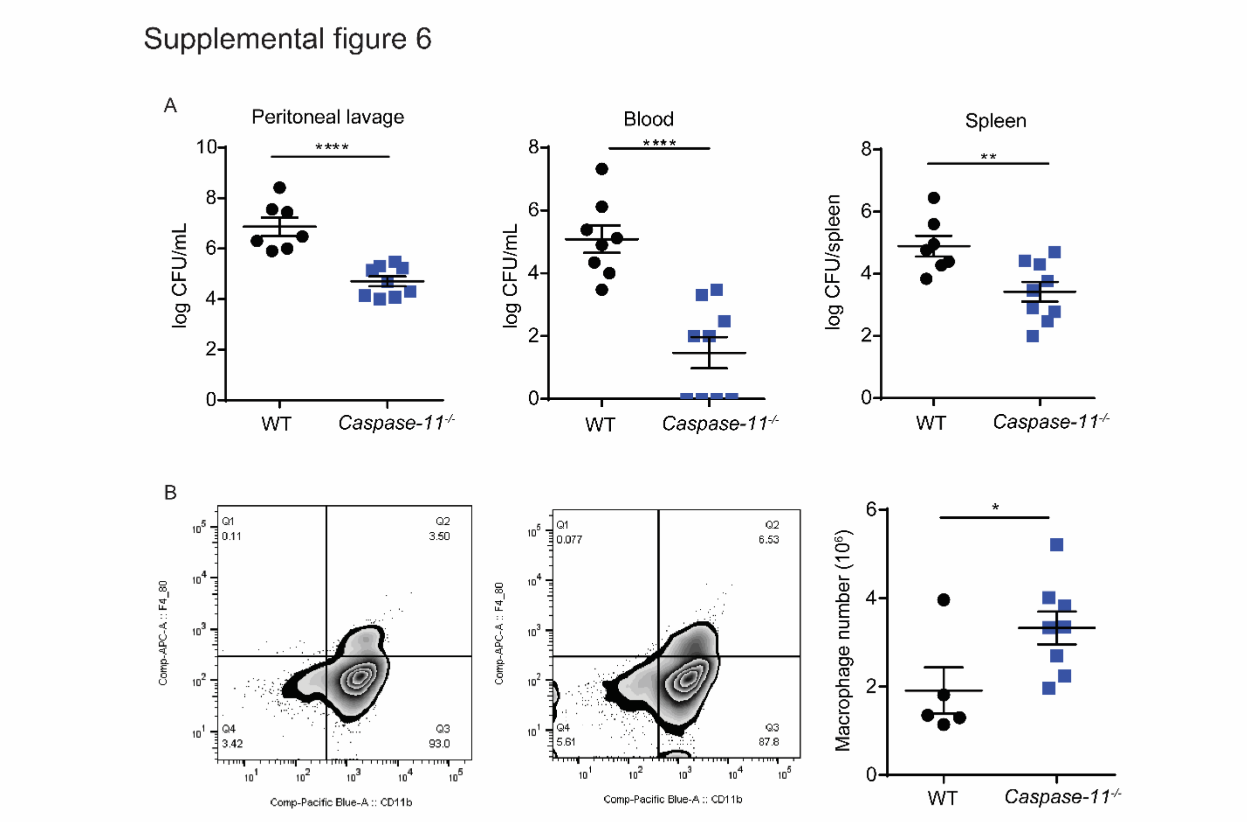

Supplemental figure 6. Absence of the *Caspase-11* gene decreases bacterial loads and increases macrophage number in mice following cecal-ligation and puncture (CLP). WT and *Caspase-11-/-* mice were subjected to CLP. (**A**) Bacterial loads in peritoneal lavage, blood and spleen 24 h after CLP (n=7-9/group). (**B**) Macrophage number in peritoneal cavity after 24 h (n=5-8/group). Graphs are expressed as mean ± SEM. Data were analyzed using Unpaired *t* test (**A, B**). *p<0.05, **p<0.01, ****p<0.0001.

Supplemental figure 7

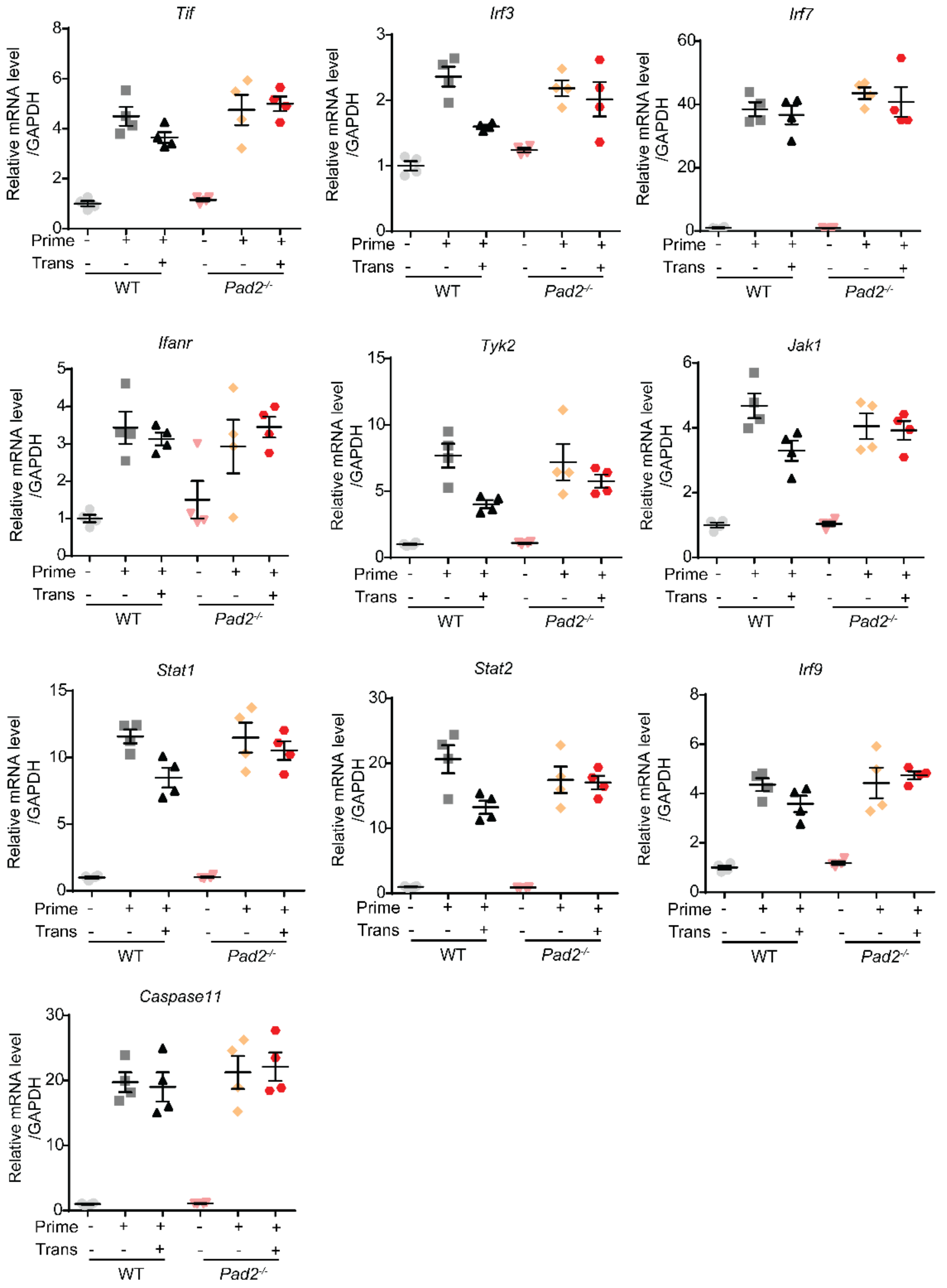

Supplemental figure 7. Absence of the *Pad2* gene does not affect gene expression in non-canonical pyroptosis pathway. Bone marrow derived macrophages from WT and *Pad2-/-* mice were treated with or without non-canonical pyroptosis stimulation. Gene expression were detected in cell lysate. Results are representative of three independent experiments. Graphs are expressed as mean ± SEM (n=4/group). Data were analyzed using two-way ANOVA with Bonferroni's multiple comparisons test.

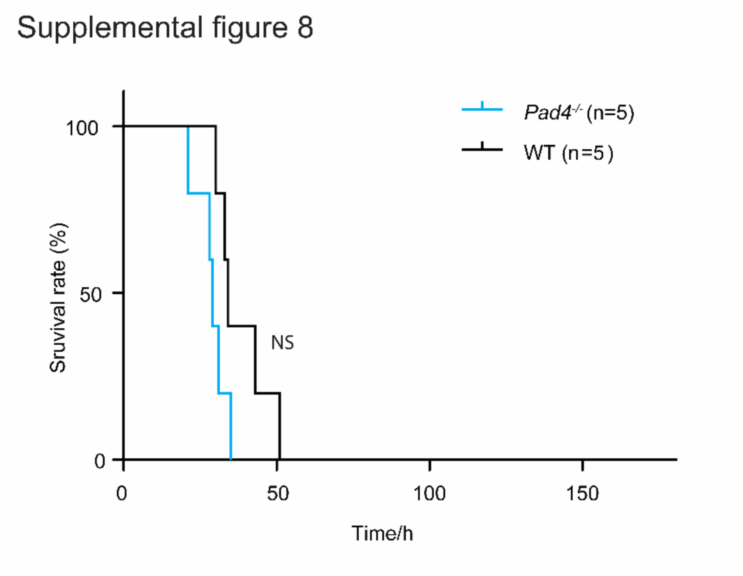

Supplemental figure 8. Absence of the *Pad4* gene does not improve survival in in mice following cecal-ligation and puncture (CLP). Kaplan-Meier survival curve of WT and *Pad4-/-* CLP mice. Values are expressed as survival percentage (n=5/group). p>0.05 by log-rank test. NS: not significant.

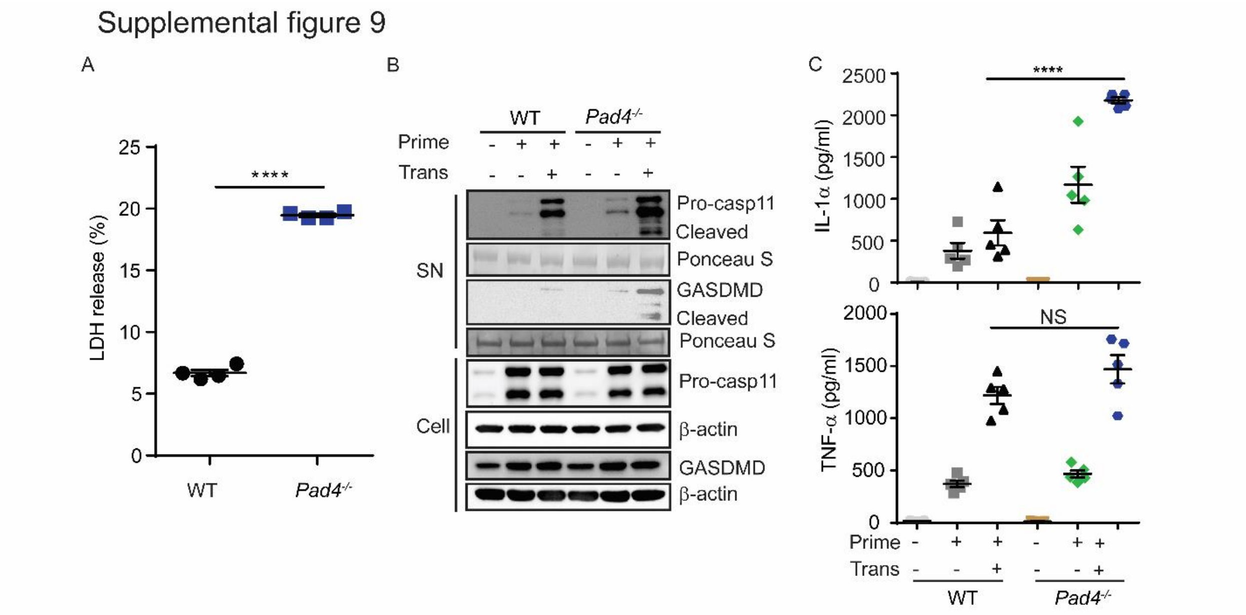

Supplemental figure 9. Absence of the *Pad4* gene enhances Caspase-11-dependent non-canonical pyroptosis in bone marrow-derived macrophages (BMDMs). (**A**) LDH release in supernatant of BMDMs after non-canonical pyroptosis treatment (n=4/group). (**B**) Representative Western blot images show Caspase-11 and GASDMD activation in supernatant and cell lysate of BMDMs with or without non-canonical pyroptosis treatment. Concentrations of (**C**) IL-1α and TNF-α in the supernatant of BMDMs with or without non-canonical pyroptosis treatment (n=5/group). Results are representative of at least three independent experiments (**A-C**). Graphs are expressed as mean ± SEM. Data with two groups were analyzed using Unpaired *t* test (**A**), and three or more groups were analyzed by two-way ANOVA analysis with Bonferroni's multiple comparisons test (**C**). *p<0.05, ***p<0.001, ****p<0.0001. SN, supernatant.

Supplemental table 1: Primer information for qPCR

Primer name	Sequence
Trif-F	CGATCAAGACGGCCATGAGTC
Trif-R	CTCGTCGGTGTCATCTTCTGC
Irf3-F	GAGAGCCGAACGAGGTTCAG
Irf3-R	CTTCCAGGTTGACACGTCCG
Irf7-F	GCGTACCCTGGAAGCATTTC
Irf7-R	GCACAGCGGAAGTTGGTCT
Ifnar-F	GACAACTACACCCTAAAGTGGAG
Ifnar-R	GCTCTGACACGAAACTGTGTTTT
Jak1-F	AGTGCAGTATCTCTCCTCTCTG
Jak1-R	GATTCGGTTCGGAGCGTACC
Tyk2-F	AGCTTCAAGAACTGCATCCCT
Tyk2-R	CACACAGGTATGCGCTCTGAG
Irf9-F	CACCATGCAAGCGAAGTATCA
Irf9-R	CGCCATTGGTCCTCCCATT
Stat1-F	TCACAGTGGTTCGAGCTTCAG
Stat1-R	CGAGACATCATAGGCAGCGTG
Stat2-F	GCATAACTTGCGAAAATTCAGCC
Stat2-R	GGATCTCACCAAGAACTCAATGT
pro-*Caspase-1*-F	TGGCAGGAATTCTGGAGCTT
pro-*Caspase-1*-R	CTTGAGGGTCCCAGTCAGTC
pro-*Caspase-11*-F	AGCGTTGGGTTTTTGTAGATGC
pro-*Caspase-11*-R	CCTTGTGAACTCTTCAGGGGA
